# Hyperconjugative aromaticity and protodeauration reactivity of polyaurated indoliums

**DOI:** 10.1038/s41467-019-13663-8

**Published:** 2019-12-10

**Authors:** Kui Xiao, Yu Zhao, Jun Zhu, Liang Zhao

**Affiliations:** 1 Key Laboratory of Bioorganic Phosphorus Chemistry and Chemical Biology (Ministry of Education), Department of Chemistry, Tsinghua University, 100084 Beijing, China; 2 State Key Laboratory of Physical Chemistry of Solid Surfaces and Collaborative Innovation Center of Chemistry for Energy Materials (iChEM), Fujian Provincial Key Laboratory of Theoretical and Computational Chemistry and Department of Chemistry, College of Chemistry and Chemical Engineering, Xiamen University, 361005 Xiamen, China

**Keywords:** Catalytic mechanisms, Reaction mechanisms

## Abstract

Aromaticity generally describes a cyclic structure composed of *sp*^2^-hybridized carbon or hetero atoms with remarkable stability and unique reactivity. The doping of even one *sp*^3^-hybridized atom often damages the aromaticity due to the interrupted electron conjugation. Here we demonstrate the occurrence of an extended hyperconjugative aromaticity (EHA) in a metalated indole ring which contains two *gem*-diaurated tetrahedral carbon atoms. The EHA-involved penta-aurated indolium shows extended electron conjugation because of dual hyperconjugation. Furthermore, the EHA-induced low electron density on the indolyl nitrogen atom enables a facile protodeauration reaction for the labile Au-N bond. In contrast, the degraded tetra-aurated indolium with a single *gem*-dimetalated carbon atom exhibits poor bond averaging and inertness in the protodeauration reaction. The aromaticity difference in such two polyaurated indoliums is discussed in the geometrical and electronic perspectives. This work highlights the significant effect of metalation on the aromaticity of polymetalated species.

## Abstract (web summary)

Hyperconjugative aromaticity combines the concepts of hyperconjugation and aromaticity and explains cyclopentadiene stability. Here, the authors demonstrate extended hyperconjugative aromaticity in a metallated indole ring, which shows extended electron conjugation due to the dual hyperconjugation.

## Introduction

**Fig. 1:**
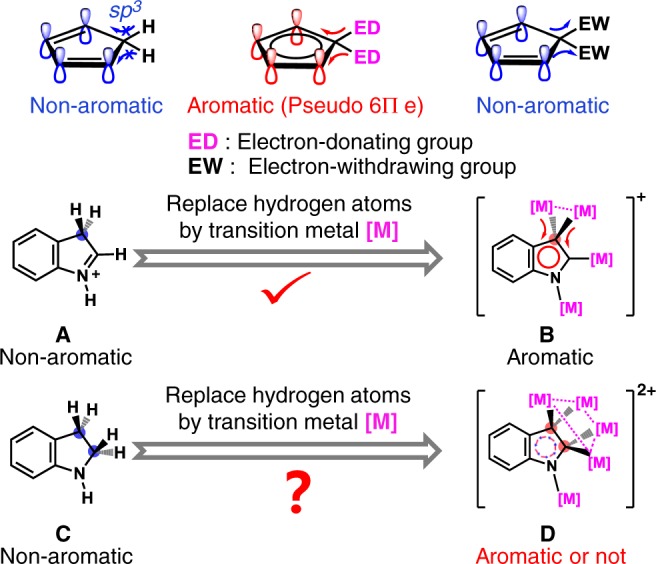
Hyperconjugative aromaticity (HA) comparison in indolium derivatives. Electron-donating groups strengthen the aromaticity in cyclopentadiene while electron-withdrawing groups act in an opposite way. Transition metal substituents in polymetalated compounds **B** and **D** give better performance on HA over the hydrogen atoms in organic models **A** and **C**.

Aromaticity is a ubiquitous and exhaustively studied concept in chemistry, which generally describes cyclic structures with significant resonance stabilization energy^1–3^. Generally, the aromatic ring is composed of *sp*^2^-hybridized carbon or hetero atoms due to the requirement of a fully conjugated π system (such as Hückel- and Möbius-π aromaticity^4–7^). The presence of a *sp*^3^-hybridized atom in the cyclic ring would severely damage the aromaticity due to an interrupted electron conjugation. Schleyer and Nyulászi demonstrated that the aromaticity of substituted cyclopentadienes 5,5-C_5_H_4_(MH_3_)_2_ (M = Si, Ge, Sn) is comparable to that of the common five-membered heteroaromatics like furan based on theoretical studies, whereas the substituted cyclopentadiene becomes antiaromatic when the substituents are changed to electron-withdrawing group (e.g. F), which act as an vacant *p* orbital^8,9^. Therein, the methylene group substituted by two electron-donating atoms could contribute pseudo 2π electrons by hyperconjugation to the four olefinic π electrons, thus leading to hyperconjugative aromaticity (HA) in cyclopentadiene with 6π electrons (Fig. 1). This finding nicely rationalizes the experimental fact that the germinal (*gem*) 5,5-isomer is the most stable one among disubstituted isomers of C_5_H_4_(MMe_3_)_2_ (M = Si and Ge)^10^. Recently, Zhu group reported that combining the negative hyperconjugation of the electron-withdrawing group (e.g. F) and push and pull effect leads to the strongest antiaromatic pyrrolium^11^. More importantly, HA helps people understand and explains some typical reactivity. For example, O’Ferrall and co-workers once correlated the HA in arenium intermediates with the enhancement of dehydration reaction rates of diols^12,13^. Recently, Houk and co-workers revealed that the reactivity and stereoselectivity of 5-substituted cyclopentadienes in Diels–Alder reactions are also related to HA^14–16^. On the other hand, Mauksch and Tsogoeva reported an alternative explanation to HA in charged sigma complexes that the topology of highest occupied molecular orbital (HOMO) of the complexes containing charged *sp*^3^ carbon is a key factor to determine the aromaticity^6^.

Thereafter, we found that transition metal substituents give better performance on HA over the traditional main-group ones^17–19^. For example, the five-membered ring (5MR) of the metalated indolium (**B**) containing a *gem*-dimetalated carbon moiety exhibits aromaticity while its parent indolium (**A**) is non-aromatic (Fig. 1). Such HA is reminiscent of the frequent appearance of *gem*-dimetalated structural units in many reported metalated aromatic intermediates^20–23^. Following this line of thinking, it is intriguing for chemists to know if the cyclic ring containing two *gem*-dimetalated *sp*^3^-hybridized atoms is still aromatic or not. This fundamental question is not only conducive to expanding our knowledge boundaries in aromaticity, but also sheds light on the mechanistic studies of related transition metal-catalyzed reactions, wherein polymetalated aromatics often act as key intermediates.

In this work, we report an extended hyperconjugative aromaticity (EHA) in a penta-aurated indolium compound and disclose its effect on the reactivity of polymetalated aromatics. In contrast to the transformation from indolium **A** to dihydroindole **C** that would lead to interrupted electron conjugation in the 5MR due to the presence of two *sp*^3^ carbon atoms (Fig. 1), we find that the introduction of two vicinal *gem*-dimetalated motifs in the polymetalated analog **D** does not impair the aromaticity. Although the penta-aurated prototype (complex **1**) of **D** arises from the addition of a gold(I) species onto the tetra-aurated indolium **2** of type **B**, structural analysis reveals that the bond averaging scenario in the 5MR of **1** is more remarkable than that of **2**. Theoretical studies show that the more extensive electron conjugation in **1** should be ascribed to dual hyperconjugation of two vicinal *gem*-diaurated motifs. Furthermore, the EHA in **1** leads to better electron conjugation in the indolyl 5MR and relatively low electron density on the indolyl nitrogen atom, enabling a facile protodeauration reaction for the labile Au-N bond. In contrast, no reaction occurs between the HA-involved **2** and the same substrate. Our finding in this work not only demonstrates the existence of aromaticity in a permetalated dihydroindole analog containing two *gem*-disubstituted sites, but also reveals the close relationship of HA with the reactivity of polymetalated aromatics.

## Results and discussion

### Synthesis and characterization of polyaurated indoliums 1 and 2

**Fig. 2:**
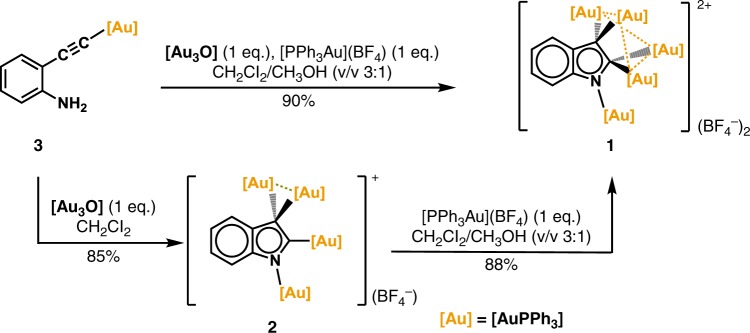
Synthetic procedures for the penta-aurated complex **1**. Both a direct and an indirect pathway are shown to highlight the intermediate role of complex **2**.

**Fig. 3:**
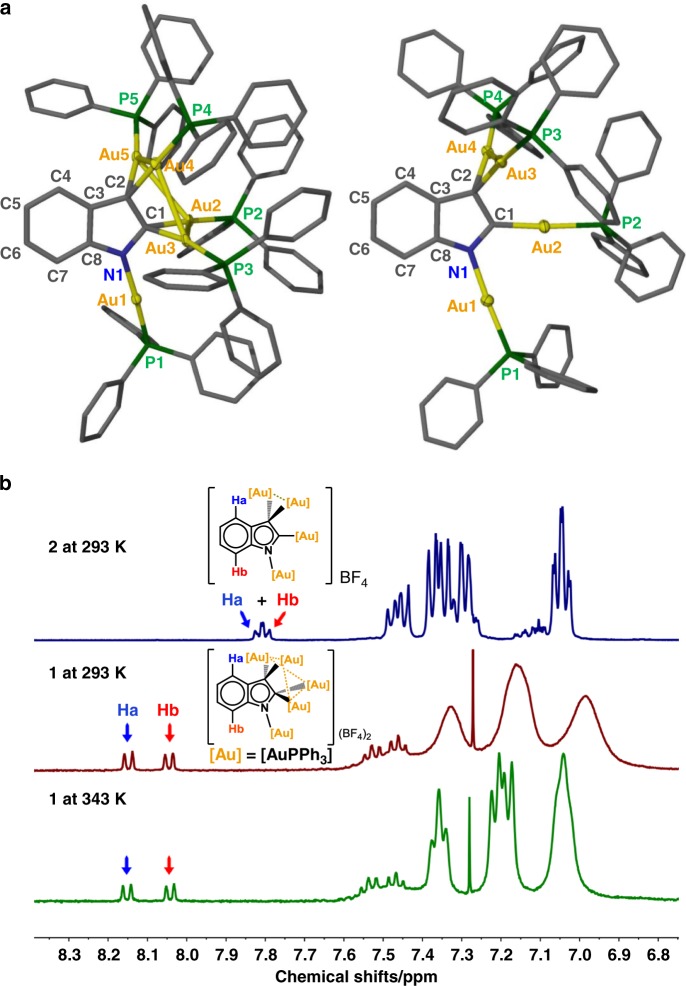
Crystal structures and ^1^H-NMR spectra of **1** and **2**. **a** Crystal structures of **1** (left) and **2** (right). Hydrogen atoms and tetrafluoroborate counter anions are omitted for clarity. Selected bond lengths (Å) and angles (°) in **1**: N1–C1 1.375(9), C1–C2 1.426(11), C2–C3 1.434(9), C3–C8 1.415(10), C8–N1 1.365(9), N1–Au1 2.062(6), C1–Au2 2.094(7), C1–Au3 2.154(6), C2–Au4 2.086(7), C2–Au5 2.140(6), ∠Au2–C1–Au3 82.6(2), ∠Au4–C2–Au5 85.0(3). **2**: N1–C1 1.316(15), C1–C2 1.466(18), C2–C3 1.435(16), C3–C8 1.442(19), C8–N1 1.368(17), N1–Au1 2.052(11), C1–Au2 2.020(11), C2–Au3 2.135(11), C2–Au4 2.096(12), ∠Au3–C2–Au4 85.5(4). **b** ^1^H-NMR spectra in *d*_*4*_-1,2-dichloroethane of **1** at 293 and 343 K, and **2** at 293 K.

The well-established gold(I) catalysis chemistry in the past decades has shown that the gold(I) ion often acts as a π-acidic center to activate the unsaturated hydrocarbon substrates by π-activation^24–26^. However, in our previous synthesis of tetra- and octa-aurated heteroaromatics by 2-ethynylaniline derivatives, the σ-activation of the ethynyl group by a gold(I) species is more essential^17^. Therein, the σ-aurated carbon–carbon triple bond is subject to the nucleophilic attack by an aurated imido group, which is formed by the reaction of a NH_2_ group with a μ_3_-oxo trinuclear gold(I) compound [(PPh_3_Au)_3_(μ_3_-O)](BF_4_) (**[Au**_**3**_**O]**)^27^, to yield the cyclized polyaurated heteroaromatics. In order to probe the possible role of the σ, π dual activation in such transformation, we purposefully added one equivalent [PPh_3_Au](BF_4_) to the 1:1 mixture of the σ-aurated compound **3** and **[Au**_**3**_**O]** (Fig. 2). The mixture was originally supposed to generate the tetra-aurated indolium complex **2**. However, the ^1^H-NMR monitoring showed the quantitative formation of complex **1** (Supplementary Fig. 1). X-ray crystallographic analysis (vide infra) revealed that **1** has a penta-aurated indolyl structure with two vicinal carbon atoms on the indolyl 5MR being *gem*-aurated. Remarkably, as an alternative synthetic pathway, complex **1** can also be directly synthesized by mixing **2** with one equivalent [PPh_3_Au](BF_4_) by aurophilic interaction, suggesting the precursor role of **2**. Complexes **1** and **2** both show good stability upon exposure to ambient air and moisture or heating up to 70 °C (Fig. 3b and Supplementary Fig. 2). Noticeably, upon elevating the temperature of the solution of **1** until decomposition, no **1**-to-**2** dissociation was detected in NMR monitoring (Fig. 3b). In addition, the calculated ∆G is −36.4 kcal mol^−1^ in the **2**-to-**1** transformation (Supplementary Fig. 3). The results jointly suggest that the complexation of **2** with a gold species may bring about extra stabilizing effect in **1**.

We further made structural comparison between **1** and **2**. Due to the poor precision of bond lengths in the previously reported crystal structure of **2** (space group *P*2_1_/*c*), we modified the purification method and re-collected the crystal data of **2** (space group *Pbca*). X-ray crystallographic analysis shows that compound **1** has a coplanar indolyl trianion skeleton, which is coordinated by five AuPPh_3_ units via a μ_5_-C1,C2,N1-η^2^,η^2^,η^1^ mode (Fig. 3a). The two carbon centers C1 and C2 in **1** each is bonded by a *gem*-digold unit with the Au–C bond lengths in the range of 2.086(7)–2.154(6) Å, which is comparable with the bond distances of the CAu_2_ unit in **2** (2.096(12) and 2.135(11) Å). In addition, the Au···Au distances and ∠Au–C–Au angles of the *gem*-digold units in **1** (2.805(1) Å and 82.6(2)° for Au2-Au3, and 2.855(1) Å and 85.0(3)° for Au4–Au5) are also similar with those of **2** (2.874(1) Å and 85.5(4)° for Au3–Au4). The Au–N bond length in **1** (2.062(6) Å) is slightly longer than that of **2** (2.052(11) Å). Despite the high similarity in the aspect of interaction between the inner indolyl skeleton and the outer gold substituents in **1** and **2**, the dimensions of the indolyl ring in these two complexes show remarkable difference. The bond length alternations (ΔBLs) of three C–C and two C–N bonds of the 5MR in **1** are 0.019(1) and 0.010(0) Å, respectively, smaller than the ΔBLs of **2** (0.031(1) Å for ΔBL_C–C_ and 0.052(2) Å for ΔBL_C–N_). The more equalized bond lengths in the 5MR of **1** indicate better electron delocalization than **2**.

The molecular composition and excellent stability of **1** in solution were confirmed by electron-spray ionization mass spectroscopy (ESI-MS) and nuclear magnetic resonance (NMR). ESI-MS exhibits two isotopically well-resolved peaks at *m*/*z* = 2497.3281 and 1205.1597 corresponding to the [**1**-BF_4_]^+^ and [**1**-2(BF_4_)]^2+^ species, respectively. ^1^H NMR spectrum of **1** reveals two doublets at 8.15 and 8.04 ppm, which can be assigned to two protons on C4 and C7 of the indolyl six-membered ring (6MR) (Fig. 3b), respectively. In contrast, the signals of the indolyl 6MR protons in **2** appear as a merged multiplet at the upfield position of 7.79 ppm. Such NMR difference should be ascribed to more remarkable electron-withdrawing effect of total five gold(I) species in **1** relative to four ones in **2**. In addition, due to the steric effect of four gathering PPh_3_ units around two vicinal carbon atoms in **1** that causes a rich variety of hardly interchangable conformations, the corresponding resonance signals of PPh_3_ in **1** are much broader than those of **2**. Upon heating from 293 to 343 K, these peaks in **1** turned into sharp due to the interconversion of different conformations (Fig. 3b).

### Theoretical investigations on the aromaticity of 1 and 2

**Fig. 4:**
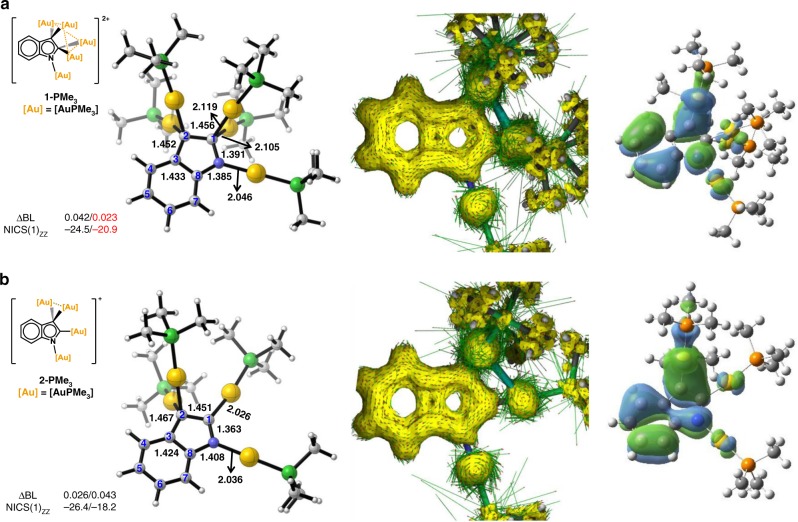
Theoretical calculation studies on 1-PMe_3_ and 2-PMe_3_. Calculated models and AICD plots (isovalue: 0.03) of the π orbitals contribution and HOMOs (isovalue: 0.02) of **a** **1-PMe**_**3**_ and **b** **2-PMe**_**3**_. The NICS(1)_*zz*_ values given before and after the ‘/’ are those computed at 1 Å above the geometrical centers of 6MR and 5MR, respectively.

In view of the remarkable structural difference of the indolyl 5MR between **1** and **2** arising from the decoration of different numbers of AuPPh_3_ units, we further carried out density functional theory (DFT) calculations to study the aromaticity of the indole rings in **1** and **2**. Model complexes **1-PMe**_**3**_ and **2-PMe**_**3**_ were simplified by replacing the PPh_3_ ligands in **1** and **2** with PMe_3_. Among many calculated descriptors applied to quantify aromaticity by criteria like energetic, structural, and electronic characteristics, the nucleus independent chemical shift (NICS)^28^ and anisotropy of the current-induced density plot (ACID)^29,30^ have gained wide recognition. In general, negative NICS values indicate aromaticity while positive ones show anti-aromaticity. As shown in Fig. 4a, b, the calculated NICS(1)_*zz*_ values of the indolyl 6MR/5MR in **1-PMe**_**3**_ and **2-PMe**_**3**_ are −24.5/−20.9 and −26.4/−18.2 ppm, respectively. This result supports the aromatic nature of the 5MR in **1-PMe**_**3**_ and **2-PMe**_**3**_. In general, introduction of a *sp*^3^-hybridized carbon atom into a ring will impair its aromaticity to some extent. When two carbon atoms in a ring become *sp*^3^-hybridized, its aromaticity might disappear. In contrast to the HA in **2-PMe**_**3**_ that has a *gem*-disubstituted moiety at the *sp*^3^-hybridized carbon atom, the unexpected aromaticity in **1-PMe**_**3**_ containing two *gem*-diaurated *sp*^3^-hybridized carbon atoms, as will be shown later, is enhanced rather than reduced by hyperconjugation of polyaurated substituents. The aromaticity in **1-PMe**_**3**_ was further confirmed by the diatropic ring current in the ACID plots (high resolution ACID graphs of **1-PMe**_**3**_ and **2-PMe**_**3**_ were shown in Supplementary Figs. 4 and 5). ACID plots are used to visualize electron delocalization in the ring, in which aromatic rings exhibit diatropic circulation whereas anti-aromatic ones show a paratropic ring current. According to the key occupied π-orbitals of **1-PMe**_**3**_ and **2-PMe**_**3**_ (Supplementary Figs. 6 and 7), both of them have five π-orbitals, leading to a 10π system. Nevertheless, accurate electron counting in metallaaromatic compounds is still puzzling and difficult^31^. Moreover, Solà and Szczepanik once pointed out that it was very challenging to unambiguously associate the aromaticity of metallaaromatics with the 4*n* + 2 (Hückel) and 4*n* (Möbius) rules because both Hückel and Möbius topology may contribute to the HOMO of some metallacycles together^32^. As shown in Fig. 4, differentiating the type of Hückel or Möbius for the key occupied π-orbitals in **1-PMe**_**3**_ and **2-PMe**_**3**_ is indeed difficult due to the low symmetry of their geometries.

In order to probe the difference of aromaticity between **1-PMe**_**3**_ and **2-PMe**_**3**_, multiple theoretical methods were applied to investigate the dissimilarity from the geometrical and electronic perspectives (Supplementary Table 1). As illustrated in the structures of **1-PMe**_**3**_ and **2-PMe**_**3**_, the ΔBL_C–C_ (0.023 Å) for the indolyl 5MR in **1-PMe**_**3**_ is smaller than that of **2-PMe**_**3**_ (0.043 Å), which is in agreement with the trend shown in the crystal structures and suggests a better electron delocalization of the EHA-involved indolyl 5MR in **1-PMe**_**3**_ than that of the common hyperconjugative aromatic **2-PMe**_**3**_. To confirm the higher electron delocalization in **1-PMe**_**3**_, we further examined the aromaticity in **1-PMe**_**3**_ and **2-PMe**_**3**_ via the methods of the electron localization function separated by the π molecular orbitals (ELF_π_)^33–35^ and the electron density of delocalized bonds (EDDB) analysis^36^. ELF_π_ maps the extent of spatial localization of π electrons in a multiple electronic system. If the difference of the bifurcation values (ΔBV(ELF_π_)) is small, the π electrons should be highly delocalized. EDDB provides an explicit degree of electronic delocalization in the π-system of the aromatic ring. Our calculations reveal that **1-PMe**_**3**_ has a smaller ΔBV(ELF_π_) (0.222) and a larger EDDB by fragment analysis (EDDB_F(*r*) = 2.663 e) in comparison with the corresponding calculated values of **2-PMe**_**3**_ (ΔBV(ELF_π_) = 0.544 and EDDB_F(*r*) = 2.085 e), demonstrating more delocalized π-electrons in the indolyl 5MR of **1-PMe**_**3**_ than those in **2-PMe**_**3**_. Compared with the traditional aromatic heteroring (Supplementary Fig. 8), the aromaticity of **1-PMe**_**3**_ is close to furan and indole. And the aromaticity of **2-PMe**_**3**_ is relatively weaker. But according to the EDDB and ELF analyses, both of them are more aromatic than the **2Au-indolium** reported in 2016 (ref. ^17^). Moreover, the aromatics induced by HA could be regulated by changing types and numbers of substituents, especially the transition metal substituents based on our previous works^11,17,18^.

We next studied the detailed bonding nature in the CAu_2_ three-membered ring of **1-PMe**_**3**_ and **2-PMe**_**3**_. As shown in the crystal structures of **1** and **2**, the C1 atom in the indolyl 5MR is *gem*-diaurated in **1**, but σ-bonded to a gold(I) species in **2**. In the computed structures, the C1–Au2 (2.105 Å) and C1–Au3 (2.119 Å) bonds in **1-PMe**_**3**_ are longer than the C1–Au2 bond in **2-PMe**_**3**_ (2.026 Å) (Fig. 4a, b), in agreement with the Mayer bond order calculation result (0.69 and 0.63 for C1–Au2 and C1–Au3 in **1-PMe**_**3**_, respectively, and 1.02 for C1–Au2 in **2-PMe**_**3**_) (Supplementary Table 2). Molecular orbital (MO) calculations reveal that the bonding orbitals of C1–Au2 and C1–Au3 bonds in **1-PMe**_**3**_ are involved in the indolyl 5MR delocalization (Supplementary Fig. 9), leading to more negative charge distribution on C1 in **1-PMe**_**3**_ (−0.335) than in **2-PMe**_**3**_ (−0.128). Furthermore, the multicenter index (MCI) value^37^ of 0.0935 for Au2–C1–Au3 in **1-PMe**_**3**_ suggests a multi-centered bonding (Supplementary Figs. 10, 11 and Table 2) in the three-membered ring. Remarkably, the region of the Au2–C1–Au3 basin in **1-PMe**_**3**_ involves more electron population (2.613 e) than that of the C1–Au2 basin (2.282 e) in **2-PMe**_**3**_ according to the basin analysis of ELF^38^, suggesting a higher electron delocalization and more charge distribution in the *gem*-digold region of **1-PMe**_**3**_ (Supplementary Fig. 12). Overall, the dual hyperconjugative effect at two vicinal carbon atoms of the indolyl 5MR enables **1** to exhibit higher delocalization and more remarkable aromaticity than **2**.

### Protodeauration reactivity of 1 and 2

**Fig. 5:**
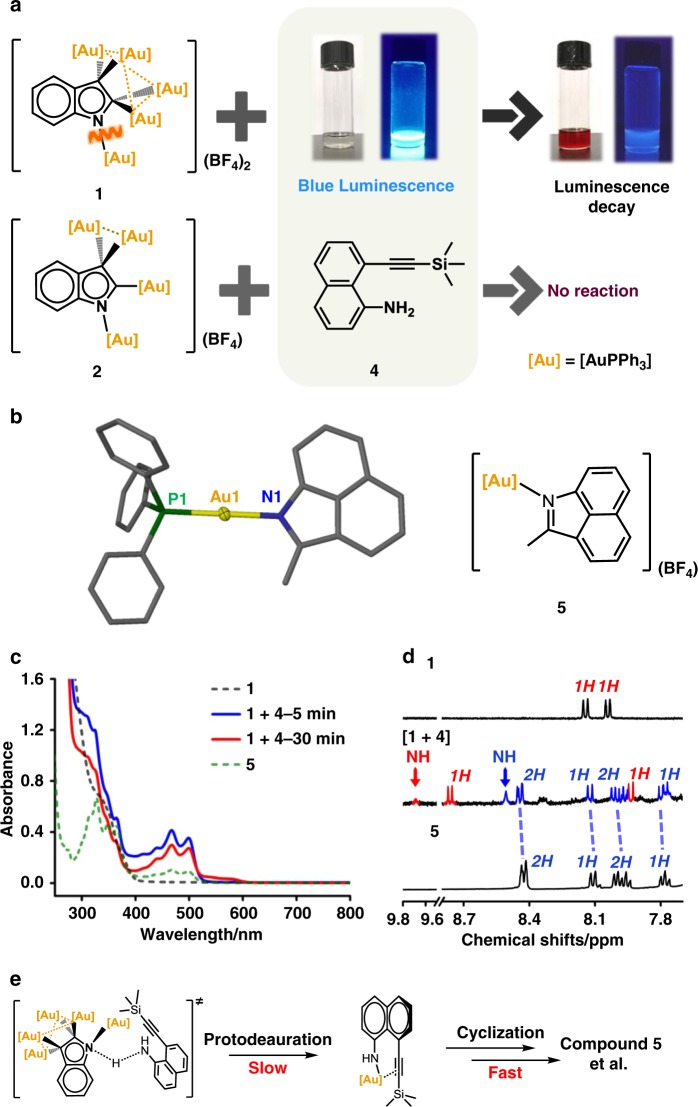
Protodeauration reactivity of 1 and 2. **a** Schematic reaction procedures of **1** and **2** with **4**. **b** Crystal structure of **5**. Hydrogen atoms and tetrafluoroborate counter anions are omitted for clarity. **c** UV-vis spectra (CH_2_Cl_2_, 298 K, *c* = 50 μM) of the reaction mixture of **1** and **4**, and complexes **1** and **5**. **d** ^1^H-NMR spectra (CD_2_Cl_2_, 298 K) of complexes **1** and **5**, and the products derived from the [**1** + **4**] mixture. **e** Proposed protodeauration step between **1** and **4**.

To gain further insight into the effect of aromaticity difference on reactivity, we then conducted the protodeauration studies of **1** and **2**. Previous investigations on gold catalysis have shown that the protodeauration reaction of organogold intermediates is the key step for catalyst regeneration and often acts as the rate-limiting step for the whole catalytic cycle^39^. Accordingly, we embarked on the protodeauration study of **1** and **2** with the 2-ethynylaniline analogs in order to reproduce the regeneration step of active gold species. Compound **4** was selected as the substrate due to its strong luminescence (quantum yield 76%) (Supplementary Table 3), which is good for reaction monitoring. As shown in Fig. 5a, we observed rapid luminescence decay upon mixing **1** with **4** while no obvious color change in the [**2** + **4**] mixture. UV-vis spectroscopic monitoring reveals the appearance of a absorption band from 400 to 520 nm upon mixing **1** and **4** (Fig. 5b). This band experienced a dramatic increase within the beginning 5 min and then a slight decrease during the next 30 min. We hypothesize that this variation arises from the formation of sequentially transformed reaction intermediates. ESI mass spectrum of the [**1** + **4**] mixture shows four strong peaks corresponding to [**1** + H-2(AuPPh_3_)-2(BF_4_)]^+^, [**1**-(AuPPh_3_)-2(BF_4_)]^+^, [**4**-H + 2(AuPPh_3_)]^+^ and [**4**-2H  + 3(AuPPh_3_)]^+^ (Supplementary Fig. 13), suggesting the possible occurrence of protodeauration for **1** and the deprotonation and auration for **4**.

To further make clear what products formed in the reaction mixture of **1** and **4**, we investigated the transformation of **4** in the presence of stoichiometric [PPh_3_Au](BF_4_). The transformation led to an organogold product **5**, which contains a benzo[cd]indole ring resulted from the nucleophilic attack of an amino group on the carbon–carbon triple bond in **4** (Fig. 5b). The nitrogen atom on the benzo[cd]indole ring in **5** is σ-bonded to a AuPPh_3_ unit, substantiating the occurrence of deprotonation for the amino group of **4** in the reaction process. Notably, the characteristic absorption band of **5** spans the range of 400–520 nm, which is similar with that of the [**1** + **4**] reaction mixture (Fig. 5c). The ^1^H-NMR monitoring reveals that the 1:1 [**1** + **4**] reaction mixture gives two sets of NMR signals (highlighted in blue and red) corresponding to the products derived from **1** and **4**, respectively (Fig. 5d and Supplementary Fig. 14). The blue NMR signals in the range of 7.7–8.5 ppm are consistent with the doublets and triplets of **5** due to six protons on the naphthalene ring. Above spectroscopic similarity with **5** suggests that the [**1** + **4**] mixture possibly undergoes a similar deprotonation and cyclization pathway to generate a mixture of benzo[cd]indole-based polyaurated complexes (Fig. 5e), which is supported by the ESI-MS spectrum (Supplementary Fig. 13). In order to make sure that the amino group in **4** indeed undergoes a deprotonated process under the treatment of **1**, we studied the reaction of **1** with a simplified model substrate aniline. The NMR monitoring showed the gradual vanishment of the characteristic doublets at 8.04 and 8.15 ppm corresponding to **1**, and the synchronous appearance of a NH singlet of indole at 9.69 ppm (Supplementary Fig. 15). Such observation is in agreement with the variation of NMR signals in the [**1** + **4**] mixture (Fig. 5d). The above experimental facts together with previous theoretical studies that reported the higher energy barriers of the *gem*-digold moieties for a protodeauration process than the unidentate ones^40,41^ suggest that the N–Au bond of **1** is the true reactive site for protodeauration.

**Fig. 6:**
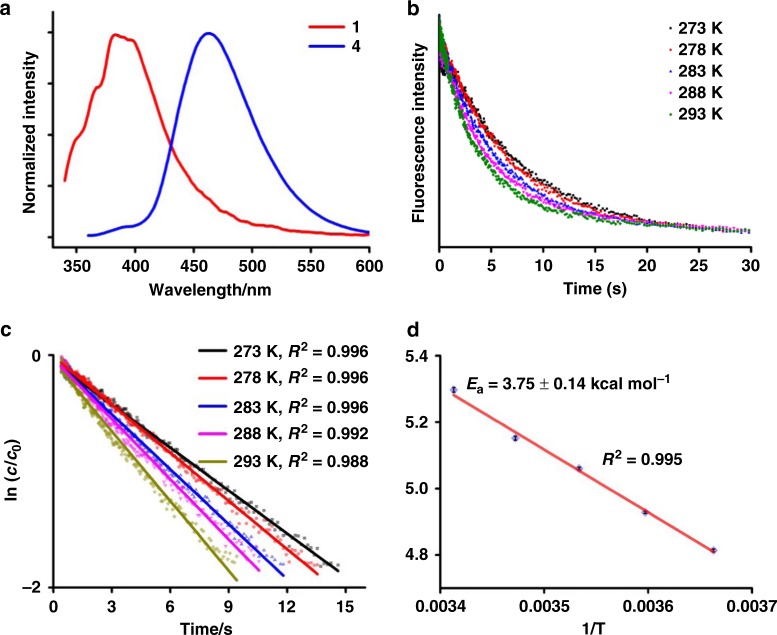
Kinetic studies on the reaction between 1 and 4. **a** Emission spectra of **1** and **4** at 298 K (excitation: 330 nm for **1** and 350 nm for **4**, *c* **=** 1.0 μM). **b** Luminescence (above 495 nm) decline curves of the reaction mixture of **1** (*c* **=** 100.0 μM) and **4** (*c* **=** 1.0 μM) in chloroform from 273 to 293 K. **c** Pseudo-first-order fitting of the luminescence decline curves. **d** Fitting with the Arrhenius equation from 273 to 293 K, the standard deviation error bars are minor than the symbols.

To further clarify the reaction details between **1** and **4**, kinetic studies based on stop-flow methods were conducted. In view of the fact that **1** and the resulting products of [**1** + **4**] like complex **5** show weak emission above 500 nm while the substrate **4** is highly luminescent at the same region (Fig. 6a and Supplementary Fig. 16), we thus monitored the decline of the luminescence above 495 nm in the stop-flow measurement. As shown in Fig. 6b, the reaction of excessive **1** with **4** is quickly finished within 30 s. The reaction acceleration upon heating from 273 to 293 K gives a good fit with a pseudo-first-order equation (Fig. 6c). According to the Arrhenius equation, the activation energy of protodeauration between **1** and **4** is estimated as 3.75 ± 0.14 kcal mol^−1^ (Fig. 6d). This activation energy is among the range of 3.1–25.8 kcal mol^−1^ for the protodeauration of organogold compounds based on previous theoretical studies^42^. We then carried out the reaction of **1** with the deuterated **4** to confirm the rate determining step of protodeauration. Hydrogen atoms of **4** were partially deuterated by mixing **4** with deuteroxide in *d*_*4*_-methanol (Supplementary Fig. 17b). Upon replacing **4** with the partially deuterated one, we observed an approximate three-fold elongation of reaction time at 273 K (Supplementary Fig. 17a). The slowdown of reaction rate due to the isotopic effect demonstrates the proton donor role of the amino group of **4** in the protodeauration step.

In sharp contrast to the remarkable reaction phenomena of **1** and **4**, no protodeauration reaction occurs between complex **2** with **4** or aniline (Supplementary Figs. 15b, 18). We subsequently probed the reasons for such distinct reactivity by computational methods. The condensed Fukui functions (f_k_^−^) (using Hirshfeld charge)^43,44^ were utilized to study different susceptibility of the N atom in **1-PMe**_**3**_ and **2-PMe**_**3**_ subject to electrophilic attack (Table 1). Generally, the atom with the largest f_k_^−^ value is the most nucleophilic site. The calculations show that the N atom in **1-PMe**_**3**_ (f_k_^−^ = 0.048) is more preferentially attacked by electrophilic reagents than **2-PMe**_**3**_ (f_k_^−^ = 0.038). In addition, the calculated BDE of the N–Au bond in **1-PMe**_**3**_ (56.5 kcal mol^−1^) in dichloromethane is weaker than that in **2-PMe**_**3**_ (63.7 kcal mol^−1^). Based on the natural population analysis (NPA)^45^, the charge distribution on the N atom in **1-PMe**_**3**_ (−0.604) is less than that in **2-PMe**_**3**_ (−0.630). This biased charge distribution should be ascribed to the dual hyperconjugative effect at both C1 and C2 of the 5MR in **1-PMe**_**3**_, which causes more electron density on C1 and the reduction of charge distribution on the N atom. Thus, the N–Au bond is elongated (2.046 Å in **1-PMe**_**3**_ relative to 2.036 Å in **2-PMe**_**3**_) and weakened, enabling a facile protodeauration reaction. In a word, the higher protodeauration reactivity of **1-PMe**_**3**_ over **2-PMe**_**3**_ should be ascribed to the weaker N–Au bond in **1-PMe**_**3**_ due to the EHA-caused high electron delocalization.

**Table 1:** Calculated data of 1-PMe_3_ and 2-PMe_3_.

	1-PMe_3_	2-PMe_3_
f_k_^−^ (N)	0.048	0.038
NPA (N)	−0.604	−0.630
NPA (Au1)	0.300	0.309
NPA (C1)	−0.335	−0.128
Bond length (N–Au)	2.046	2.036
Bond order (N–Au)	0.77	0.82
BDE in CH_2_Cl_2_ (N–Au)	56.5	63.7

Calculated condensed Fukui function (f_k_^−^), NPA analysis on N, Au1 and C1 atoms, bond length (Å), Mayer bond order and bond dissociation energy (BDE, kcal mol^−1^) of the N–Au bond.

In conclusion, the present work shows that the 5MR in the penta-aurated indolium **1** with two *gem*-digold groups exhibits better electron delocalization and HA than that of the tetra-aurated indolium **2** containing one *gem*-digold group. As a result, the NPA charge density of the nitrogen atom and the BDE value of the N–Au1 in **1** are smaller than **2**. Such difference in aromaticity accounts for the easy occurrence of protodeauration for **1**. These findings not only promote better understanding on the concept of HA, but also open bright prospects in the future development of polymetallic reactions depending on HA.

## Methods

### General information

All commercially available reagents were used as received. The solvents used in this study were dried by standard procedures. [O(AuPPh_3_)_3_]BF_4_](**[Au**_**3**_**O]**)^27^ and 8-Indonaphthalen-1-amine^46^ were prepared according to the published methods. Complexes **2** and **3** were synthesized according to our previous work^14^. ^1^H-, ^13^C-, ^31^P-NMR and H-H COSY were carried out on a JEOL ECX-400 MHz instrument. H-C HMBC were carried out on a Bruker Avance 600 MHz instrument. The UV light irradiation experiment was carried out using Agilent Cary Series UV-vis-NIR. High-resolution mass spectra were obtained on a Thermo Scientific Exactive Orbitrap instrument with an ESI mode. Stopped-flow measurements were carried out using Applied Photophysics Chirascan SX20 Stopped-Flow Spectrometer. ^1^H-, ^13^C-, ^31^P-NMR, H-H COSY, H-C HMBC and high-resolution mass spectra are shown in Supplementary Figs. 19–35.

### Synthesis of complex 1

A methanol solution (1 mL) of AgBF_4_ (6.62 mg, 0.034 mmol) was mixed with a CH_2_Cl_2_ solution (1 mL) of PPh_3_AuCl (16.83 mg, 0.034 mmol). The mixture was stirred at r.t. for 10 min. The supernatant was collected by centrifugation and was then added into a CH_2_Cl_2_ (2 mL) solution of **3** (19.56 mg, 0.034 mmol) and **[Au**_**3**_**O]** (50.35 mg, 0.034 mmol). The mixture gradually turned into light yellow after 5 min stirring. After removing the solvent by rotary evaporation under vacuum, the residual was re-dissolved in CH_2_Cl_2_ (1 mL), which was added dropwise into 30 mL petroleum ether under vigorous stirring. A white precipitate was collected by filtration. Yield: 90% (79 mg, 0.030 mmol). Single crystals of **1** were obtained by vapor diffusion of diethyl ether into a CHCl_3_ solution of **1**. ^1^H-NMR (400 MHz, CD_2_Cl_2_): *δ* 8.18–8.12 (d, *J* = 8.2 Hz, 1H), 8.08–8.01 (d, *J* = 7.8 Hz, 1H), 7.51–7.11 (m, 52H), 7.09–6.92 (s, 25H). ^13^C-NMR (100 MHz, CD_2_Cl_2_): *δ* 134.2, 134.1, 133.9 133.7, 132.9, 132.4, 130.0, 129.7, 129.5, 128.5, 128.0, 125.0, 123.0 and 117.0. ^31^P-NMR (162 MHz, CD_2_Cl_2_): *δ* 35.41, 32.33. HR-MS (ESI): calcd. for [**1**-BF_4_]^+^ (Au_5_BC_98_F_4_H_79_NP_5_) 2496.3288, found 2496.3281, calcd. for [**1**–2(BF_4_)]^2+^ (Au_5_C_98_H_79_NP_5_) 1205.1625, found 1205.1597. Elemental analysis: calcd. for (Au_5_B_2_C_98_F_8_H_79_NP_5_) C, 45.55; H, 3.08; N, 0.54. Found: C, 45.57; H, 3.10; N, 0.71.

### Synthesis of the substrate 4

8-Indonaphthalen-1-amine (250 mg, 0.929 mmol), CuI (16.9 mg, 0.089 mmol) and Pd(PPh_3_)_2_Cl_2_ (39.2 mg, 0.056 mmol) were dispersed in 30 mL triethylamine. The mixture in a 100-mL flask was degased by N_2_ for three times. Then trimethylsiylacetylene (1.3 mL, 9.30 mmol) was added under inert atmosphere. The dark mixture was stirred overnight at r.t. After removing the solvent by rotary evaporation, the residual was purified by silica gel column, eluted with a mixed solution of petroleum ether and ethyl acetate (100:5 in volume). Yield: 90% (200 mg, 0.836 mmol, yellow oil). ^1^H-NMR (400 MHz, CD_2_Cl_2_): *δ* 7.70 (d, *J* = 7.9 Hz, 1H), 7.56–7.52 (m, 1H), 7.30–7.19 (m, 2H), 7.17–7.12 (m, 1H), 6.65 (dd, *J* = 7.4, 2.4 Hz, 1H), 5.55 (s, 2H), 0.30–0.23 (s, 9H). ^13^C-NMR (101 MHz, *d*_6_-DMSO) *δ* 145.85, 135.63, 132.96, 131.04, 127.93, 125.17, 120.91, 117.02, 116.86, 110.55, 107.77, 100.29, 0.10. HR-MS (ESI): calcd 240.1201 for (C_15_H_18_NSi)^+^, found 240.1203.

### Synthesis of complex 5

A methanol solution (1 mL) of AgBF_4_ (8.14 mg, 0.042 mmol) was mixed with a CH_2_Cl_2_ solution (1 mL) containing PPh_3_AuCl (20.69 mg, 0.042 mmol). The mixture was stirred at r.t. for 10 min. The supernatant was collected by centrifugation then added into a CH_2_Cl_2_ solution containing **4** (10 mg, 0.042 mmol) in a vial. The mixture gradually changed from light yellow to orange within 2 min under stirring. After removing the solvent by rotary evaporation under vacuum, the residual was re-dissolved in CH_2_Cl_2_ (1 mL), which was then added dropwise into 30 mL petroleum ether under vigorous stirring. The orange precipitate was collected by filtration. Single crystals of **5** were obtained by vapor diffusion of diethyl ether into a CHCl_3_ solution of **5**. Yield: 80% (24 mg, 0.034 mmol). ^1^H-NMR (400 MHz, CD_2_Cl_2_): *δ* 8.42 (dd, *J* = 7.6, 2.1 Hz, 2H), 8.11 (dd, *J* = 8.3, 1.8 Hz, 1H), 8.03–7.93 (m, 2H), 7.81–7.76 (m, 1H), 7.70–7.46 (m, 15H), 3.13 (d, *J* = 1.8 Hz, 3H). ^13^C-NMR (100 MHz, CDCl3): *δ* 135.6, 134.2, 134.1, 132.9, 130.2, 129.9, 129.8, 129.2, 122.2 and 77.6. ^31^P-NMR (162 MHz, CD_2_Cl_2_) δ 31.54. HR-MS (ESI): calcd. for [**5**-BF_4_]^+^ (C_30_H_24_AuNP) 626.1306, found 626.1295. Elemental analysis: calcd. for (C_30_H_24_AuBF_4_NP): C, 50.36; H, 3.28; N, 2.03. Found: C, 50.26; H, 3.33; N, 1.96.

### X-ray crystallographic analysis

Single-crystal X-ray data for complexes **1**, **2** and **5** were collected respectively at 100 K, 173 K and 107 K with Cu-Kα radiation (*λ* = 1.54178 Å) on a Rigaku Saturn 724/724 + CCD diffractometer with frames of oscillation range 0.5°. The selected crystal was mounted onto a nylon loop in poly-isobutene and immersed in a low-temperature stream of dry nitrogen gas during data collection. All structures were solved by direct methods, and non-hydrogen atoms were located from difference Fourier maps. Non-hydrogen atoms were subjected to anisotropic refinement by full-matrix least-squares on F^2^ using the SHELXTL program and Olex^2^ program^47^. All Figures were processed by using X-seed program. The details of X-ray crystallographic measurements are summarized in the Supplementary Methods.

### Computational details

The calculated models (**1-PMe**_**3**_ and **2-PMe**_**3**_) were optimized at the TPSS^48^ level of DFT with the dispersion corrections described by EmpiricalDispersion = GD3(BJ)^49^ in the gas phase with 298.15 K and 1.00 Atm. The cartesian coordinates are shown in Supplementary Data 1. The suitability of the method mentioned above was already tested in previous works^17^. The structures without imaginary frequency indicates they have arrived the minima of all stationary points. The Au atom was described by ECP60MDF basis set^50^ and other atoms (C, H, N and P) were described by the standard 6–31 G(d) basis set^51^ at all calculations which were carried out in Gaussian 09 package^52^. The BDE energy of N–Au bonds in solvent was the single-point energy with SMD model^53^. The calculations of MCI, ELFπ and condensed Fukui Functions were carried out by Multiwfn package^54^. The orbitals chosen to ELFπ calculation of **1-PMe**_**3**_ are HOMO, HOMO-1, HOMO-4, HOMO-10, HOMO-28, HOMO-44 and HOMO-65, and for **2-PMe**_**3**_, they are HOMO, HOMO-1, HOMO-3, HOMO-9, HOMO-10, HOMO-21, HOMO-25, HOMO-37 and HOMO-42. NPS was calculated by NBO 6.0 program^45^. The geometry of models was visualized by CYL view software^55^.

## Supplementary information

Supplementary Information

Description of Additional Supplementary Files

Supplementary Data 1

## Data Availability

The X-ray crystallographic coordinates for structures reported in this article have been deposited at the Cambridge Crystallographic Data Centre (CCDC), under deposition number CCDC-1922291(**1**), 1922290(**2**) and 1936578(**5**). These data can be obtained free of charge from the Cambridge Crystallographic Data Centre via www.ccdc.cam.ac.uk/data_request/cif. For full characterization data including NMR and ESI-MS spectra and experimental details, see the Supplemental Information. Any further relevant data are available from the authors upon reasonable request.
