# Supplementary material for: Hyperconjugative aromaticity and protodeauration reactivity of polyaurated indoliums
Source: Nat Commun. 2019 Dec 10;10:5639. doi: 10.1038/s41467-019-13663-8 (PMC6904676; doi:10.1038/s41467-019-13663-8)
Supplement: Supplementary file 1 — Supplementary Information [file 41467_2019_13663_MOESM1_ESM.pdf]

Supplementary Information

Effect of gem-diaurated pattern on aromaticity and protodeauration reactivity in polyaurated indoliums

Xiao et al.

Supplementary Figures

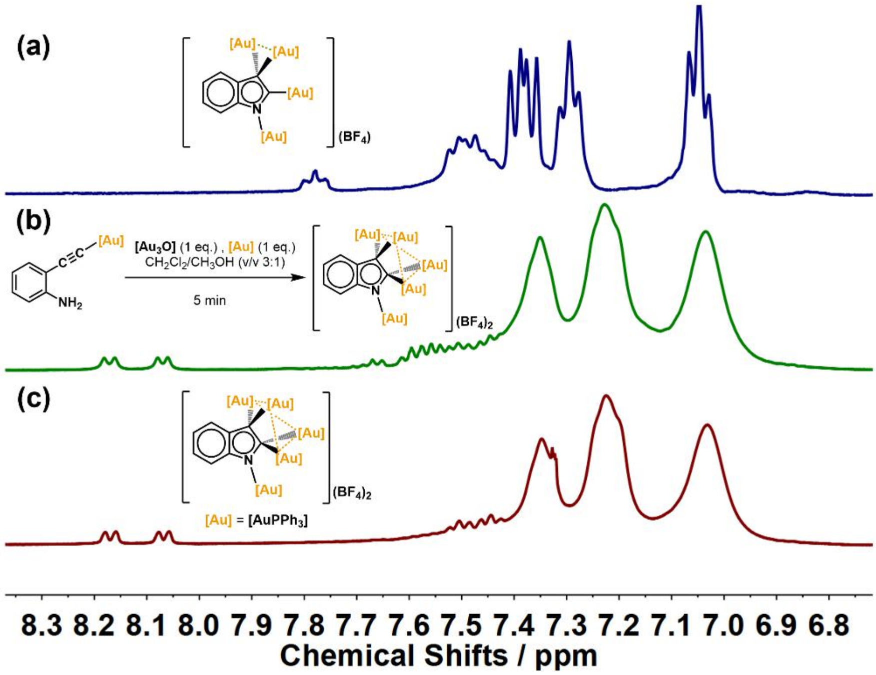

Supplementary Figure 1 ^{1}H NMR spectra (400 MHz, CD_2Cl_2, 298 K) of complex **1** and **2**. (**a**) complex **2**, (**b**) the reaction mixture for the **3**-to-**1** transformation and (**c**) complex **1**.

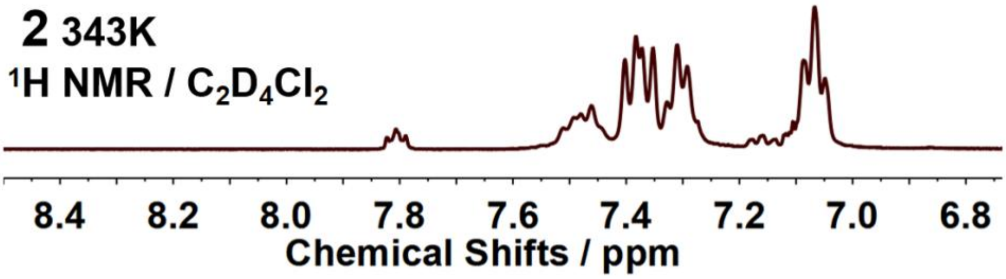

Supplementary Figure 2 ^{1}H-NMR spectrum of **2** at 343K (400 MHz, d_4-1,2-dichloroethane, 343 K).

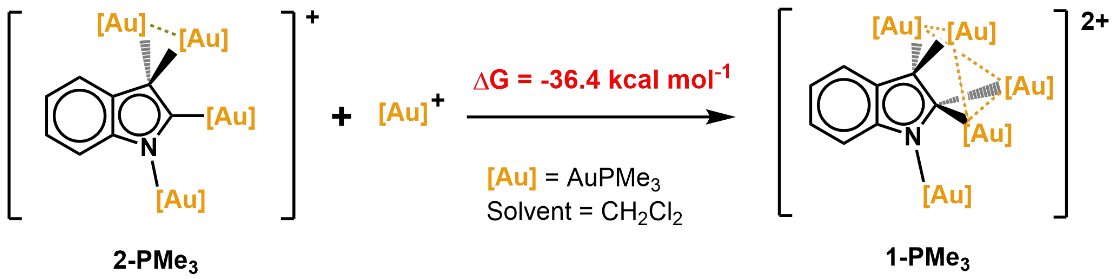

Supplementary Figure 3 The calculated thermodynamic value for the complex **2**-to-**1** transformation.

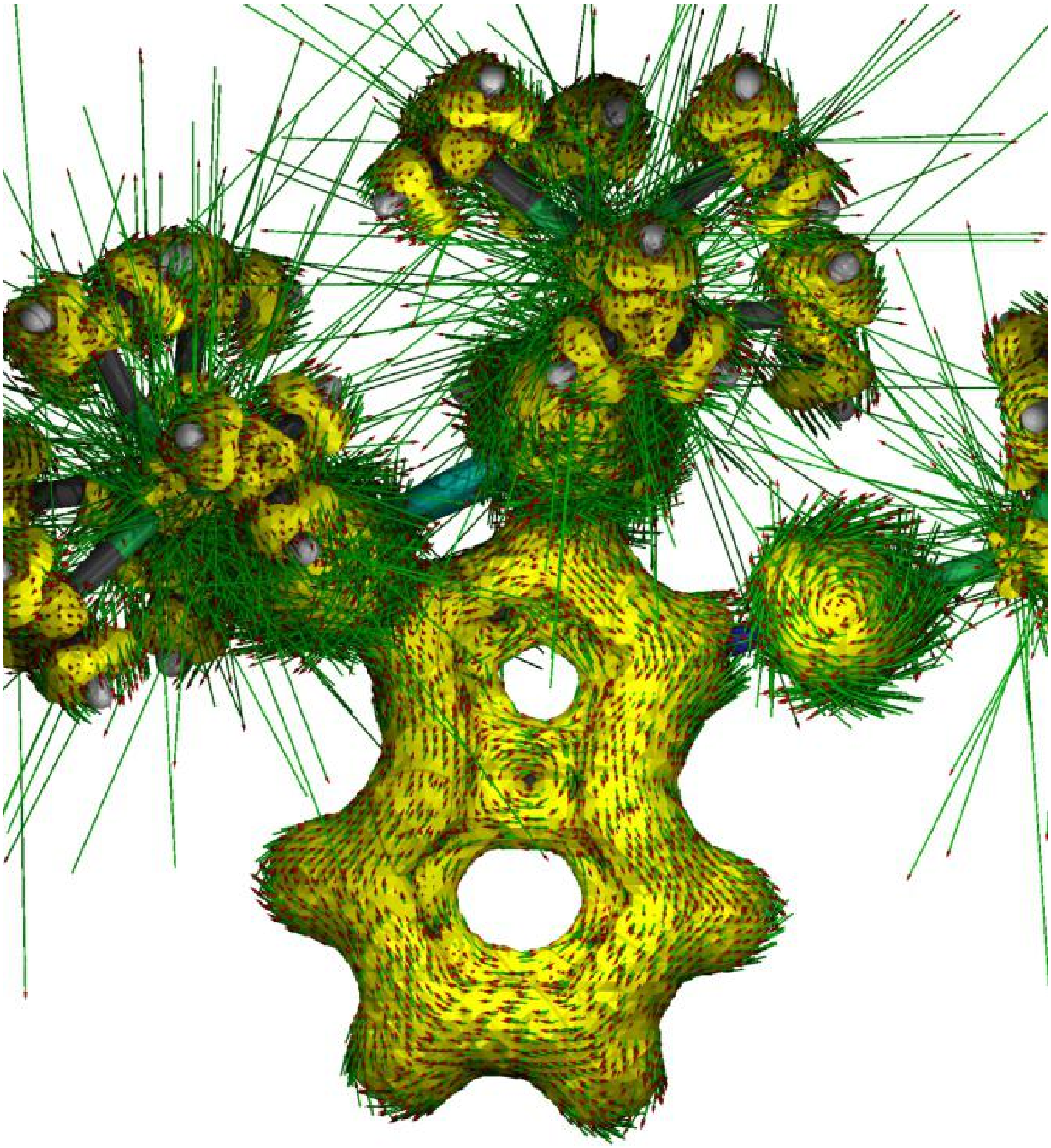

Supplementary Figure 4 ACID graph of **1-PMe3**. The magnetic field vector is orthogonal with the ring plane and point upward. The clock wise current density vectors indicate diatropic ring current (Isovalue 0.030).

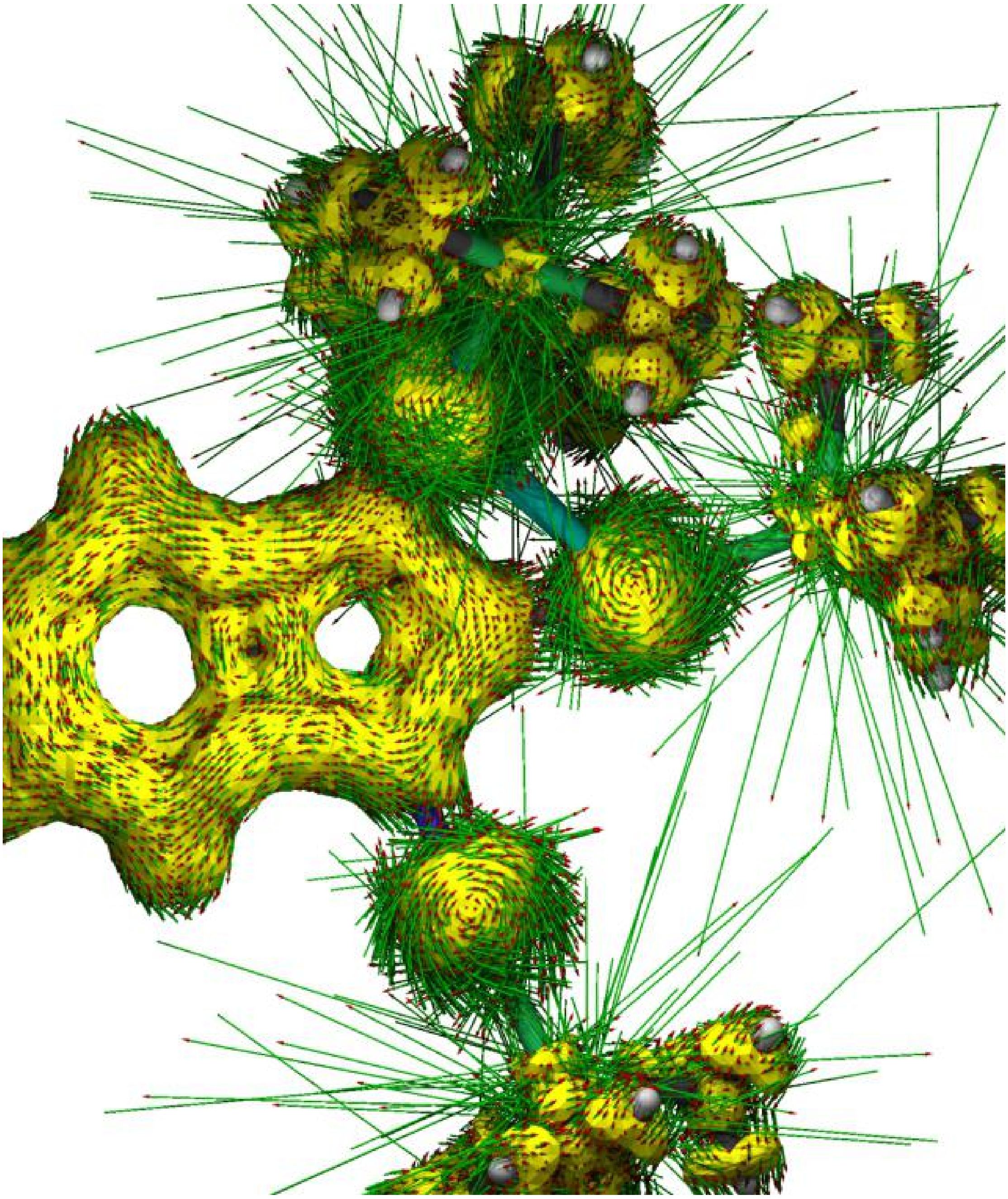

Supplementary Figure 5 ACID graph of **2-PMe$_3$**. The magnetic field vector is orthogonal with the ring plane and point upward. The clock wise current density vectors indicate diatropic ring current (Isovalue 0.030).

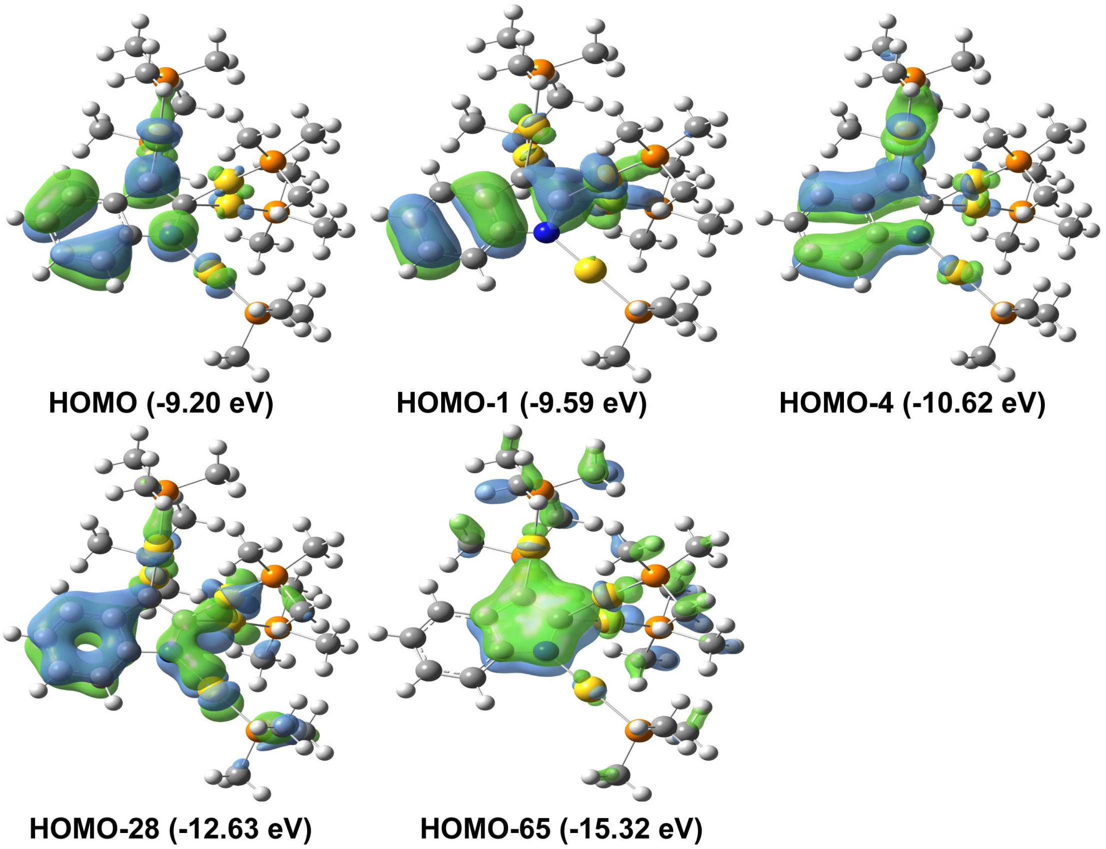

Supplementary Figure 6 The selected key π orbitals with the sum of contribution from nine atoms on the ring larger than 40% of **1-PMe$_3$** (isovalue: 0.03).

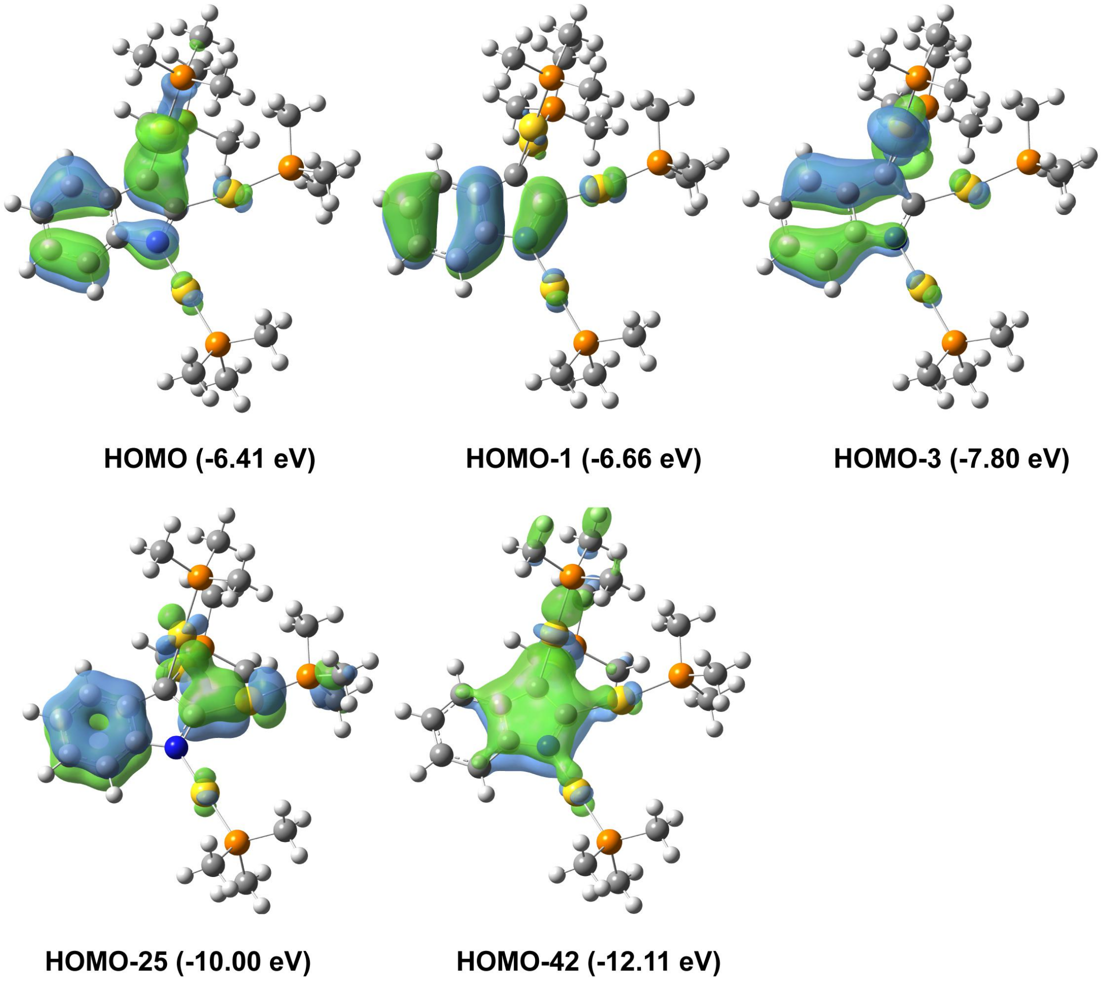

Supplementary Figure 7 The selected key π orbitals with the sum of contribution from nine atoms on the ring larger than 40% of **2-PMe$_3$** (isovalue: 0.03).

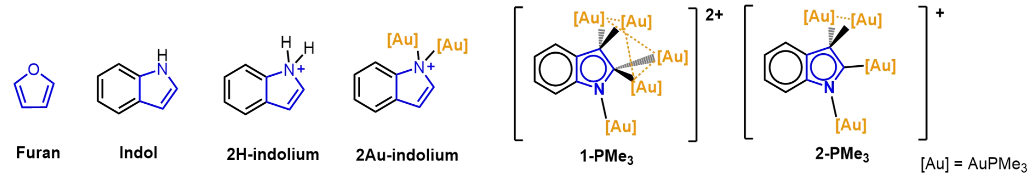

	Furan	Indol	2H-indolium	2Au-indolium	1-PMe$_3$	2-PMe$_3$
NICS(1)$_{zz}$	-26.4	-28.7	-3.6	-19.6	-20.9	-18.2
$\Delta BL_{C\text{-}C}$	0.071	0.062	0.119	0.082	0.023	0.043
EDDB_F(r)	2.879	2.397	0.513	1.427	2.663	2.085
$\Delta BV(ELF_{\pi})$	0.527	0.399	0.995	0.663	0.222	0.544

Supplementary Figure 8 The aromaticity comparison. The blue marks denote the compared five-membered ring. NICS calculation (ppm), bond length difference between C-C bonds ($\Delta BL_{C\text{-}C}$ Å), EDDB_F(r) and $\Delta BV(ELF_{\pi})$ analysis were performed to estimate their aromaticity.

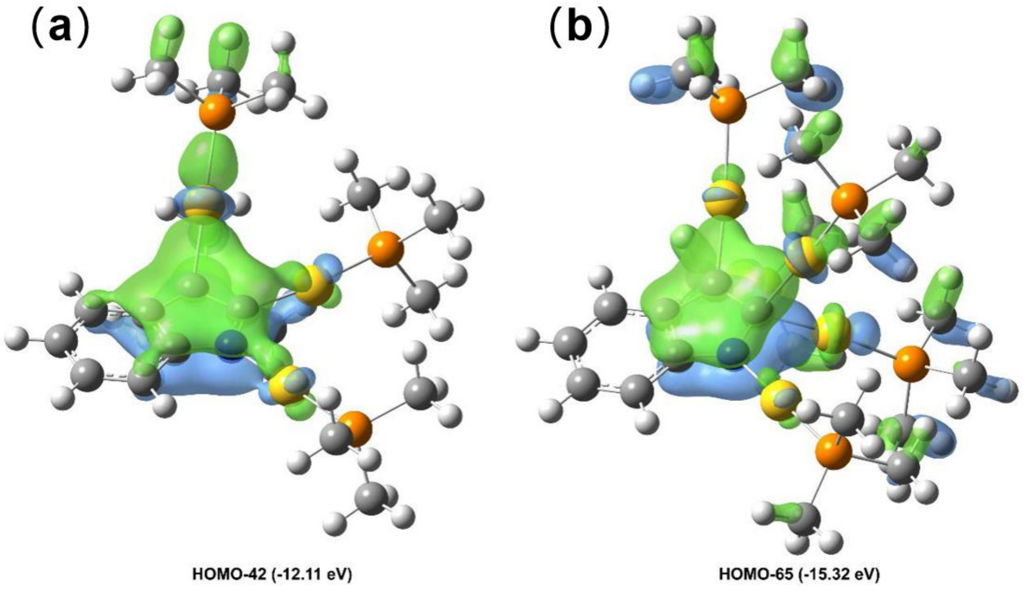

Supplementary Figure 9 Molecular orbitals of the 5MR delocalization. (**a**) HOMO-42 orbital of **2-PMe$_3$**. (**b**) HOMO-65 orbital of **1-PMe$_3$** (isovalue: 0.03).

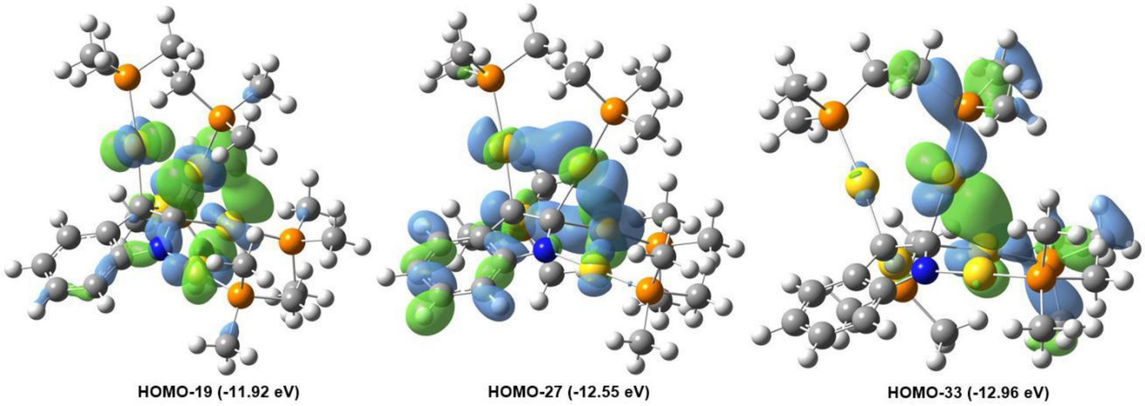

Supplementary Figure 10 Molecular orbitals related to the aurophilicity between Au2 and Au3 atoms of **1-PMe$_3$** (isovalue: 0.03).

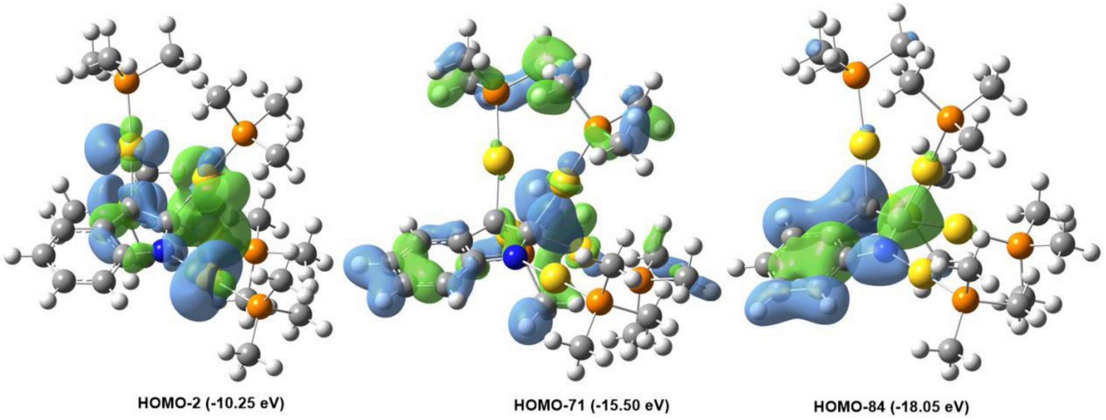

Supplementary Figure 11 Molecular orbitals related to multi-center bonding of Au2-C1-Au3 region in **1-PMe₃** (isovalue: 0.03).

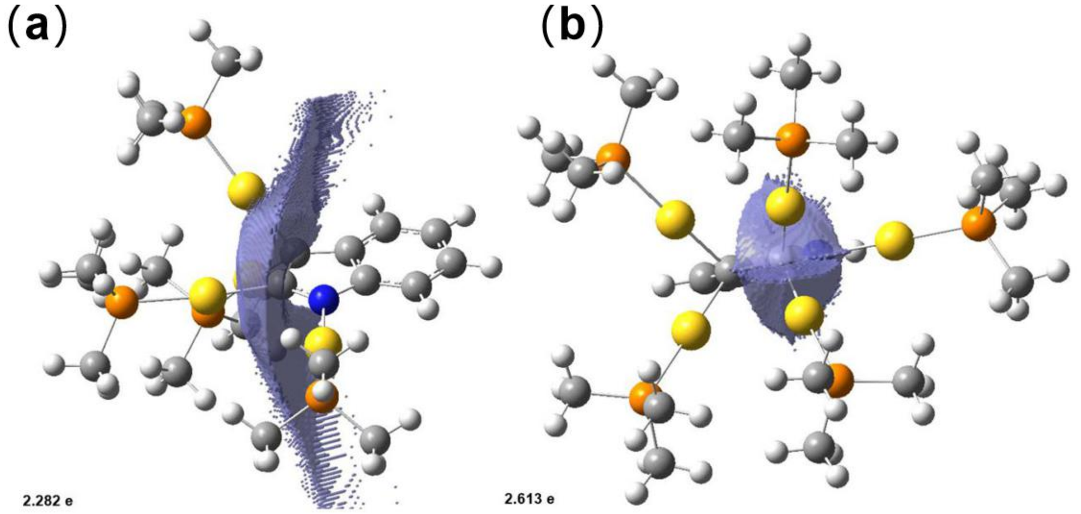

Supplementary Figure 12 Basin analysis. (**a**) The basin V(Au2, C1) of **2-PMe₃**. (**b**) The basin V(Au2, C1, Au3) of **1-PMe₃**.

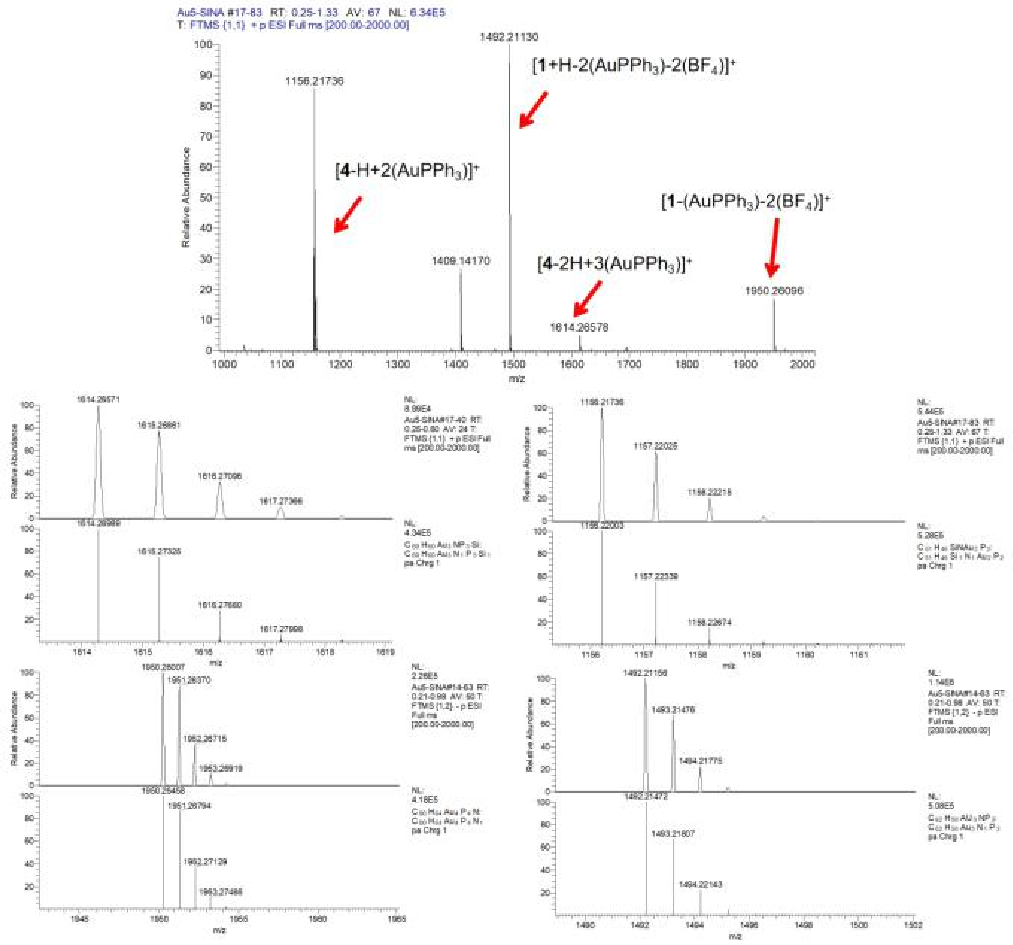

Supplementary Figure 13 ESI-MS spectra of the product of **1** and **4** ([**1**] : [**4**] = 1:1).

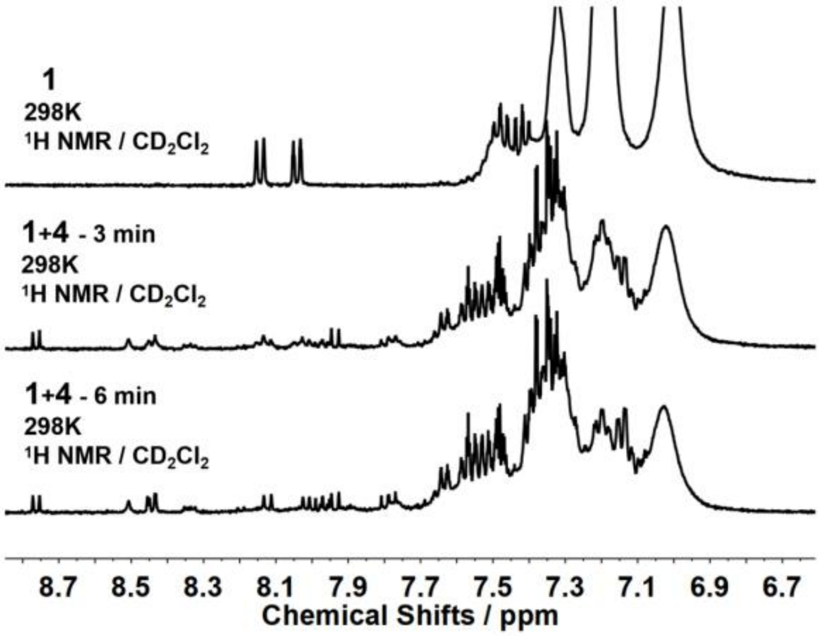

Supplementary Figure 14 ^{1}H-NMR spectra of the reaction mixture of **1** and **4** in CD_2Cl_2 at 298K ([**1**] : [**4**] = 1:1).

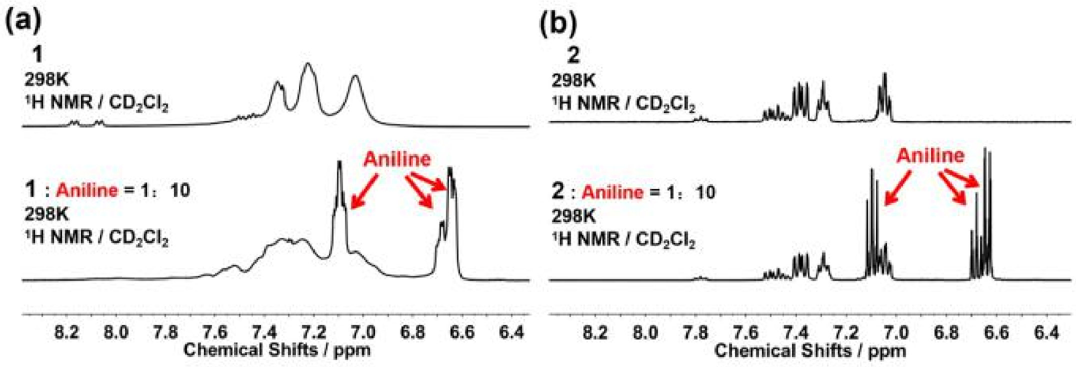

Supplementary Figure 15 ^{1}H-NMR spectra of the reaction mixture. (**a**) [**1**+aniline], (**b**) [**2**+aniline] at 298K ([**1**] : [aniline]/ [**2**] : [aniline] = 1:10).

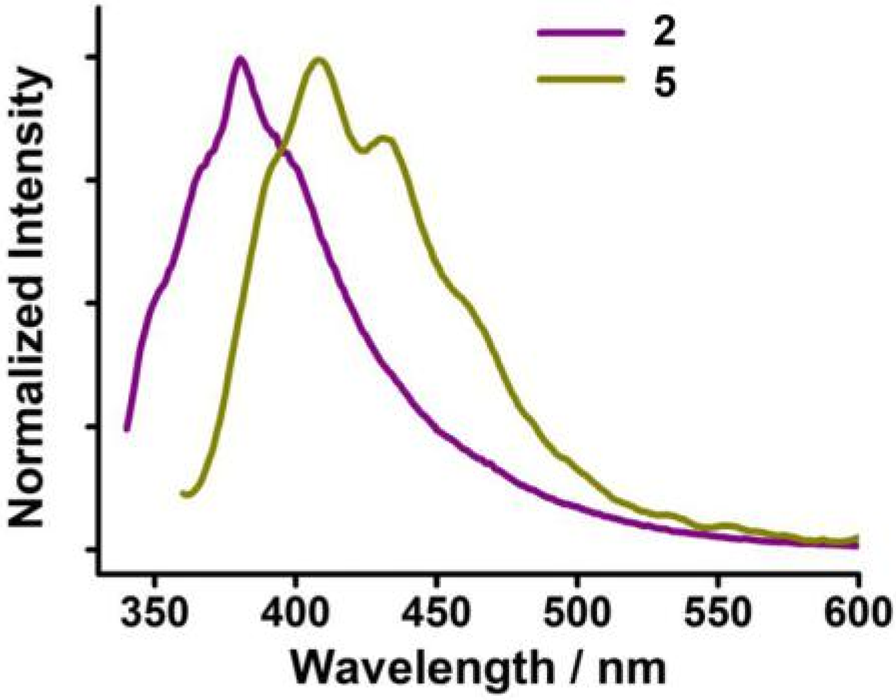

Supplementary Figure 16 Fluorescence emission spectra of **2** and **5** at 298K (Excitation: 330nm for **2**, 350nm for **5**) ([**2**] = [**5**] = 1 μM).

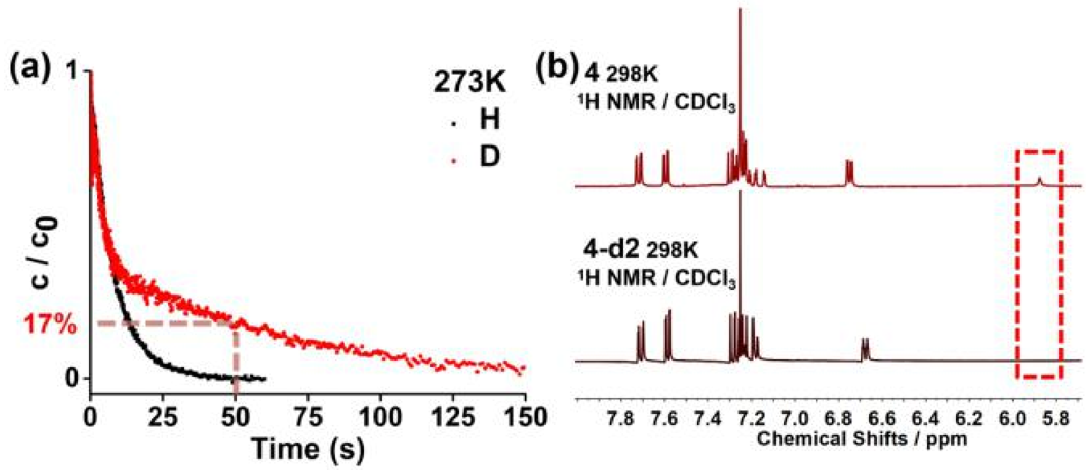

Supplementary Figure 17 Kinetic studies on isotope effect. (**a**) Fluorescence decline process of the reaction solution of **1** and deuterated **4** in chloroform in 150s at 273K.(The fast decay in the first 5s could be ascribed to the hydrogen deuterium exchange between the deuterated amino and chloroform solvent)[1] [**1**] = 100 μM, [**4**] = 1 μM, (Condition: λ_{ex} = 350 nm, Bandwidth = 2.0 nm, Trigger: External, Filter: 495nm. The timebase are 60s for **4** and 300s for deuterated **4**. Here are 500 points per measurement for **4** and 1000 points per measurement for deuterated **4**). (**b**) ^{1}H-NMR spectra of the deuterated **4** in $CDCl_3$ at 298K.

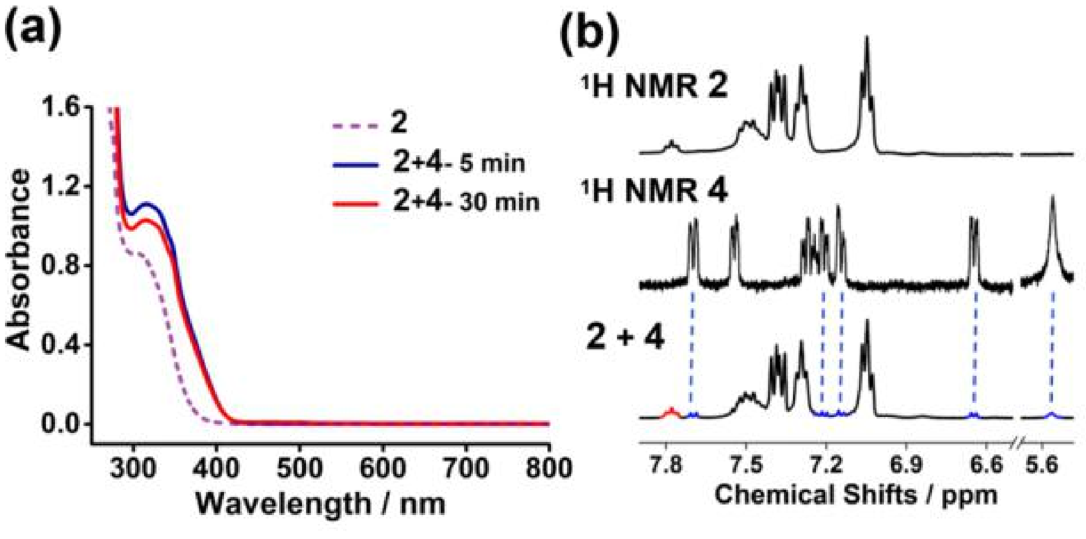

Supplementary Figure 18 Reaction studies of **2** and **4**. (**a**) UV-vis spectra of the reaction process of **2** and **4** in dichloromethane at 298K ([**2**] = [**4**] = 50 μM). (**b**) ^{1}H-NMR spectra o f the mixture of [**2** + **4**] in CD_2Cl_2 at 298K ([**2**] : [**4**] = 1:1).

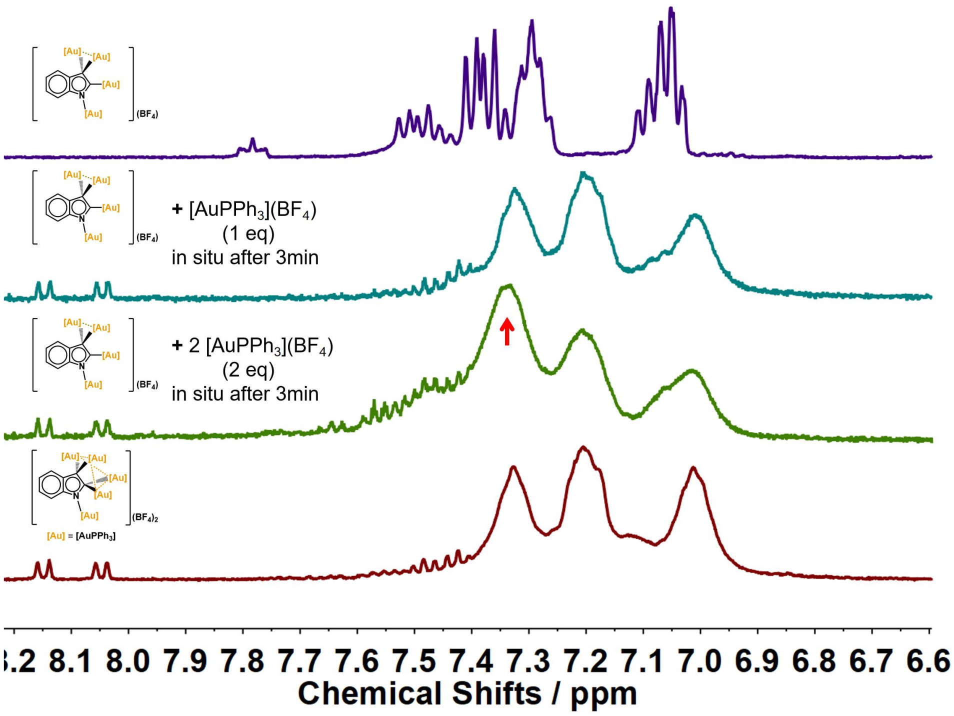

Supplementary Figure 19 *In situ* ^{1}H NMR spectra of **2**-to-**1** (400 MHz, CD_2Cl_2, 298K).

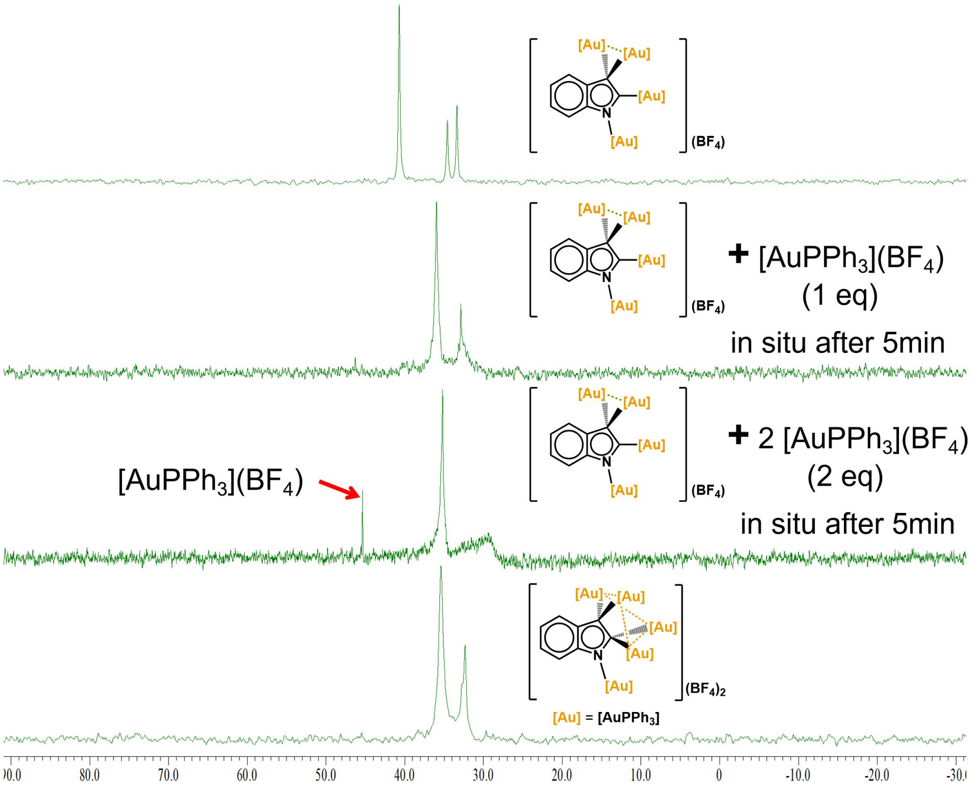

Supplementary Figure 20 *In situ* ^{31}P NMR spectra of **2**-to-**1** (162 MHz, CD_2Cl_2, 298K).

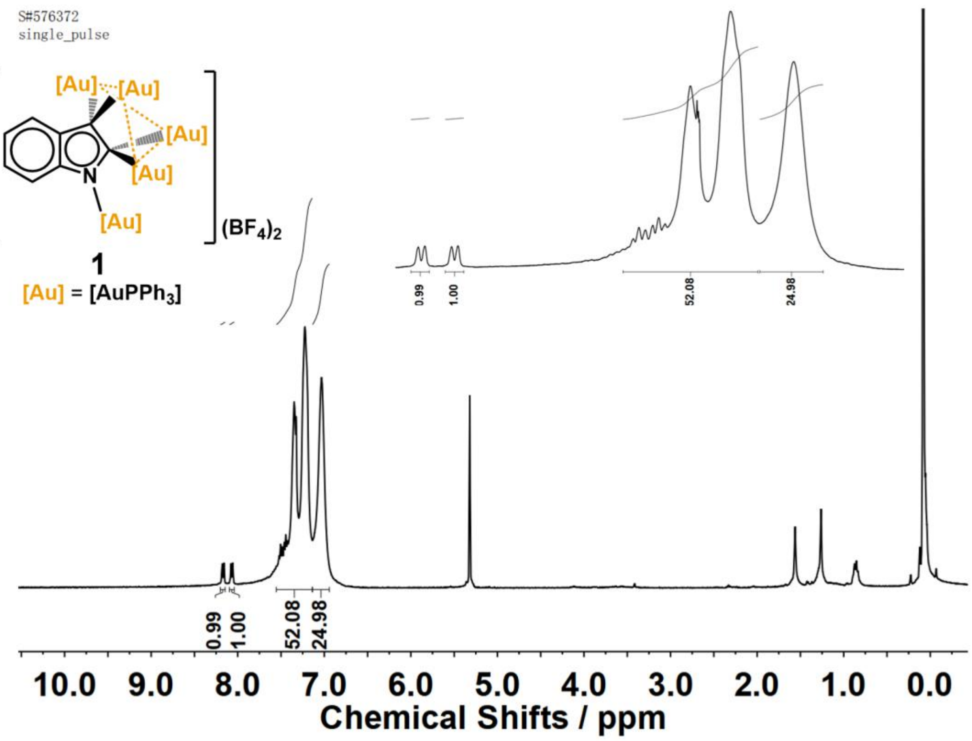

Supplementary Figure 21 ^{1}H NMR spectrum of **1** (400 MHz, CD_2Cl_2, 298K).

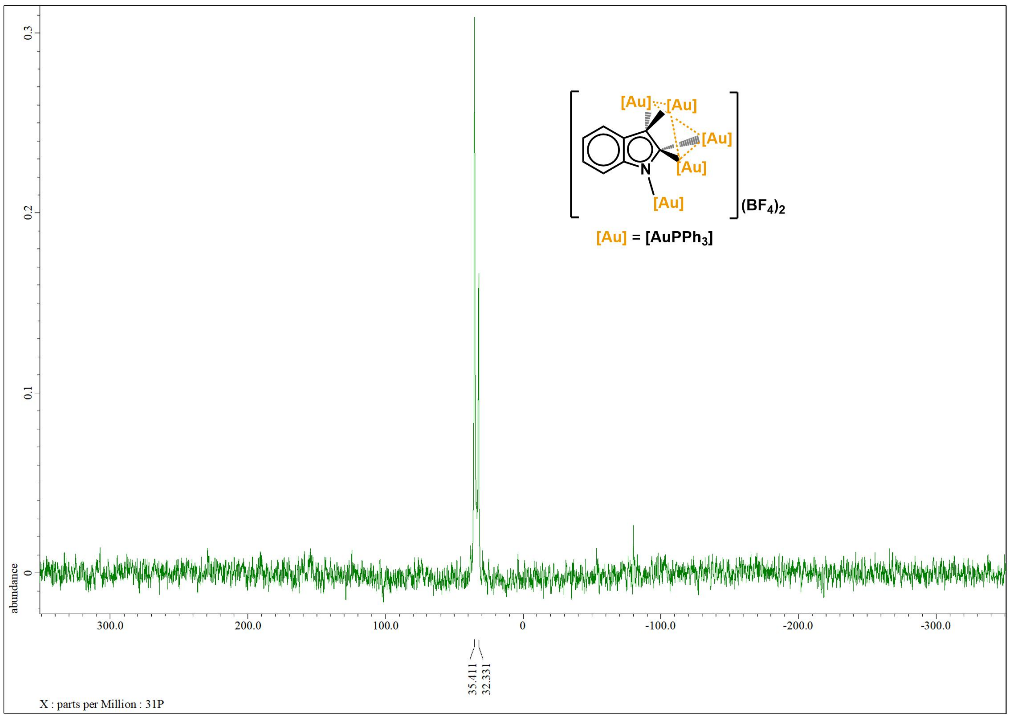

Supplementary Figure 22 ^{31}P NMR spectrum of **1** (162 MHz, CD_2Cl_2, 298K).

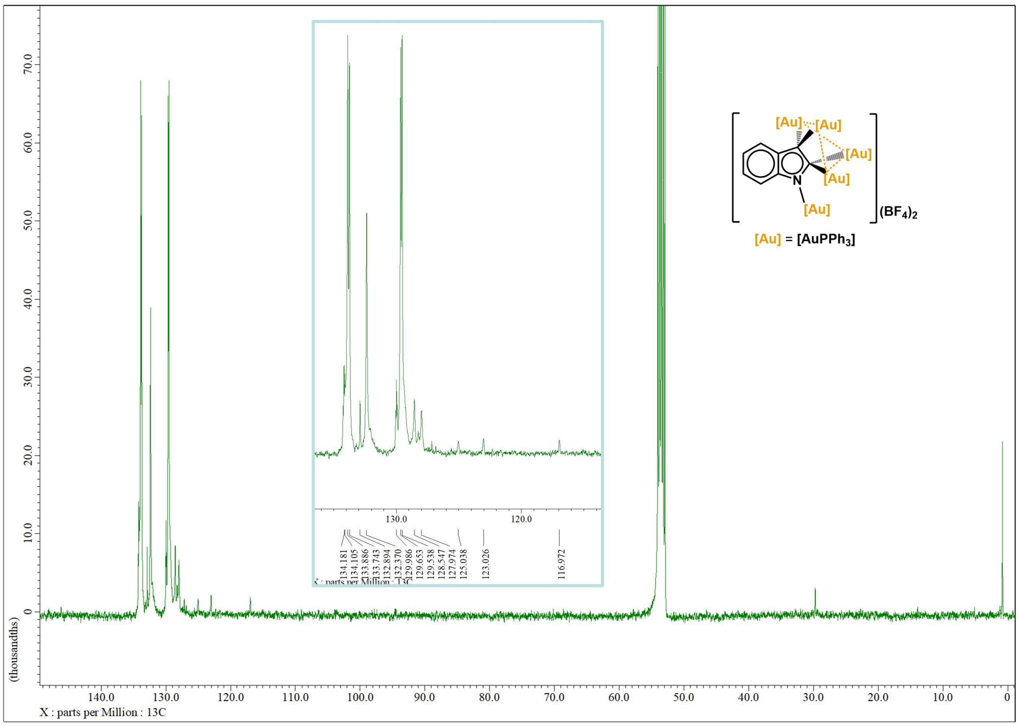

Supplementary Figure 23 ^{13}C NMR spectrum of **1** (100 MHz, CD_2Cl_2, 298K).

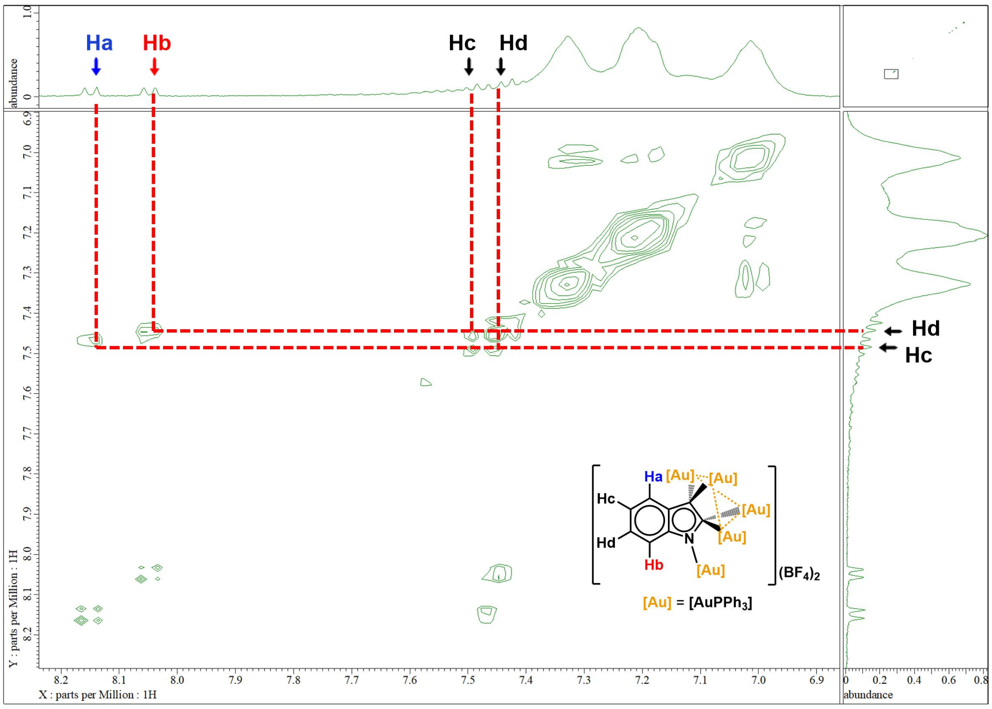

Supplementary Figure 24 H-H COSY spectrum of **1** (400 MHz, CD_2Cl_2, 298K).

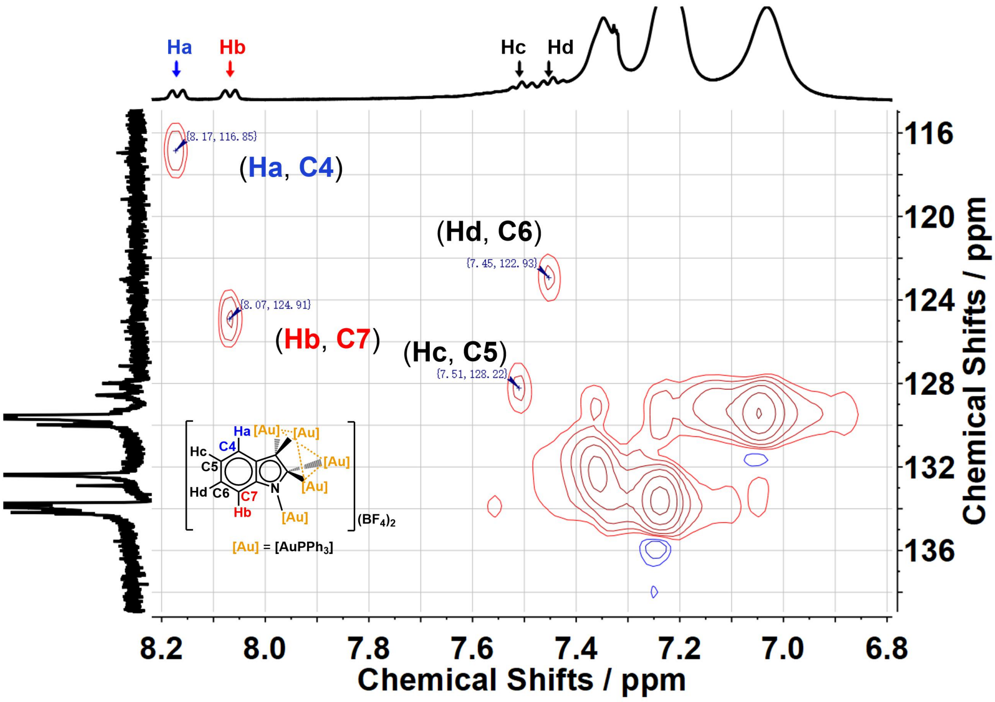

Supplementary Figure 25 H-C HMBC spectrum of **1** (600 MHz, CD_2Cl_2, 298K).

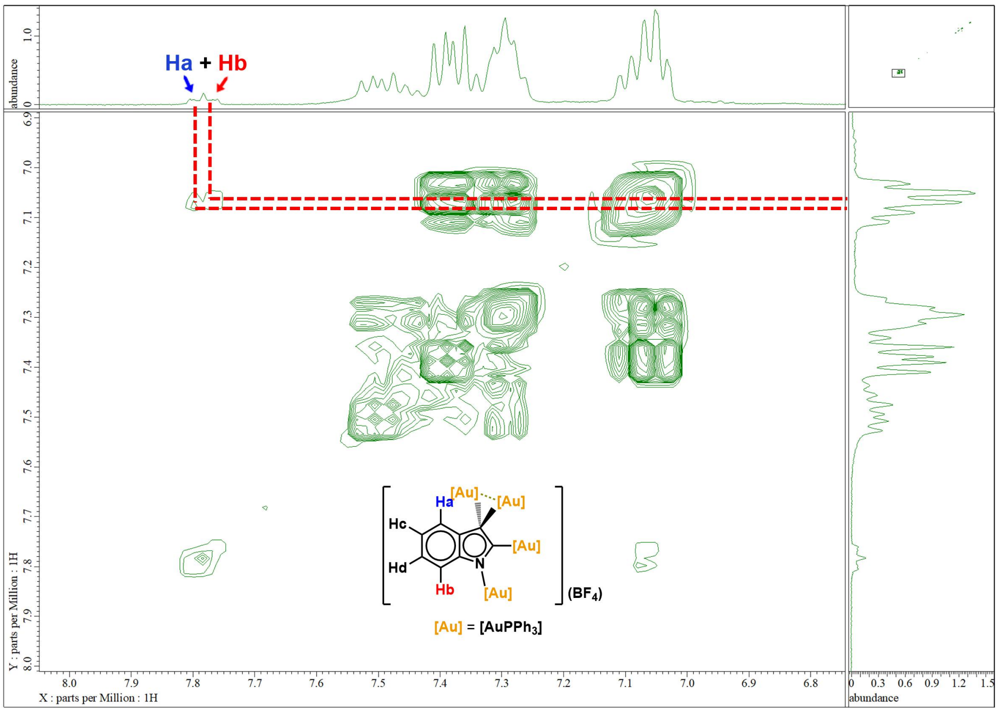

Supplementary Figure 26 H-H COSY spectrum of **2** (400 MHz, CD_2Cl_2, 298K).

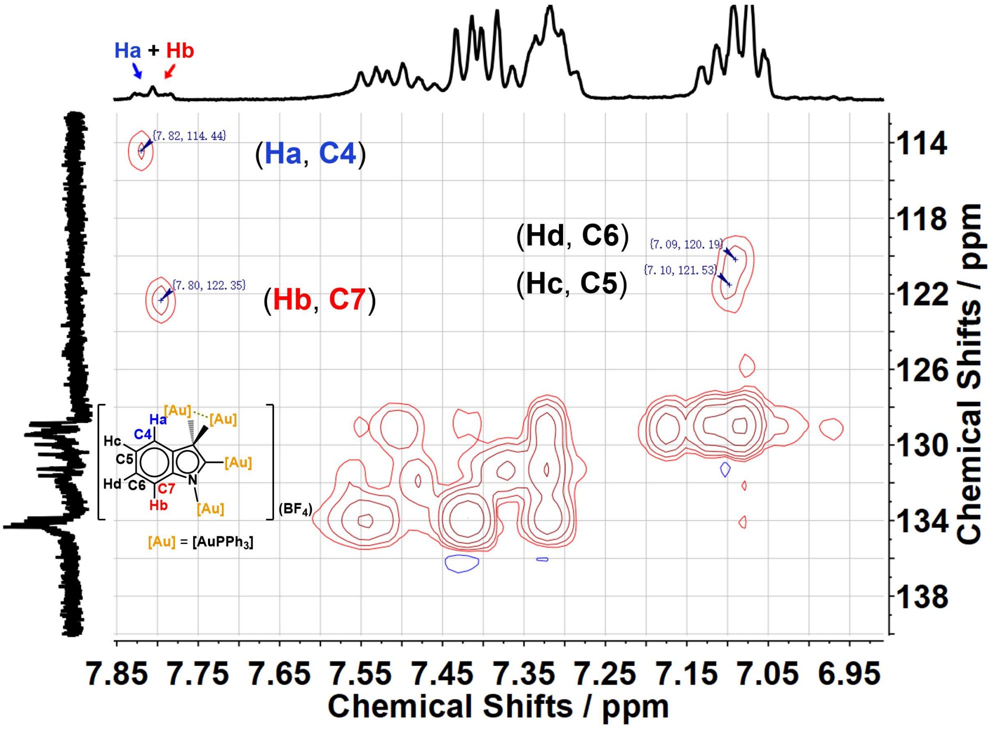

Supplementary Figure 27 H-C HMBC spectrum of **2** (600 MHz, CD_2Cl_2, 298K).

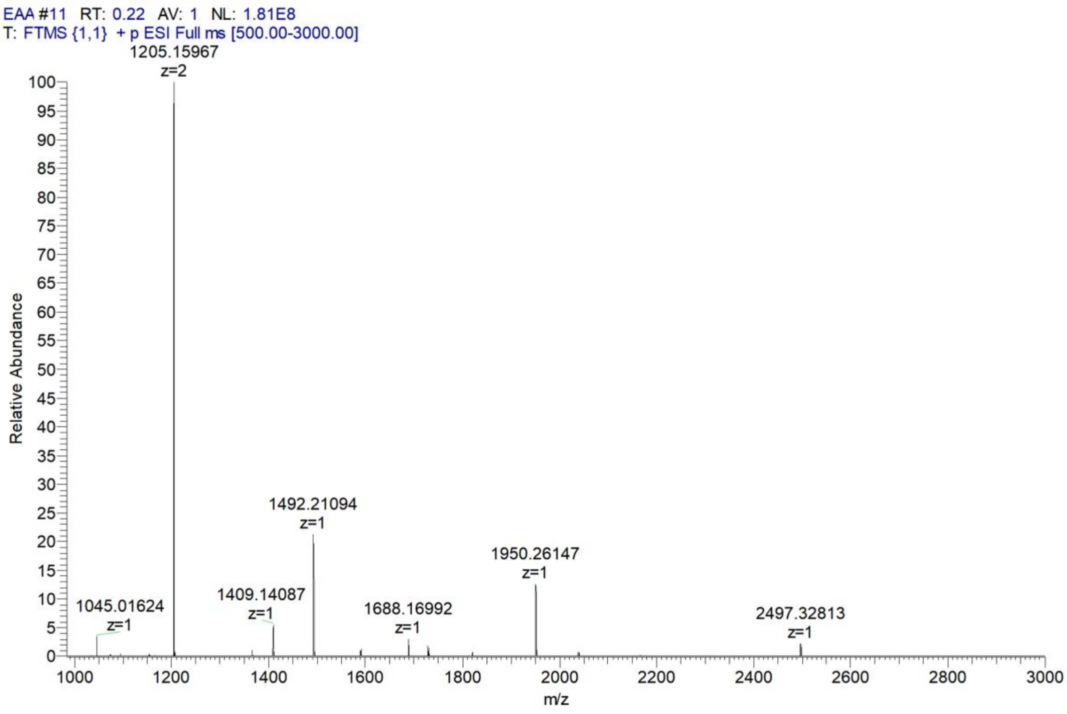

Supplementary Figure 28 ESI-MS spectrum of **1**.

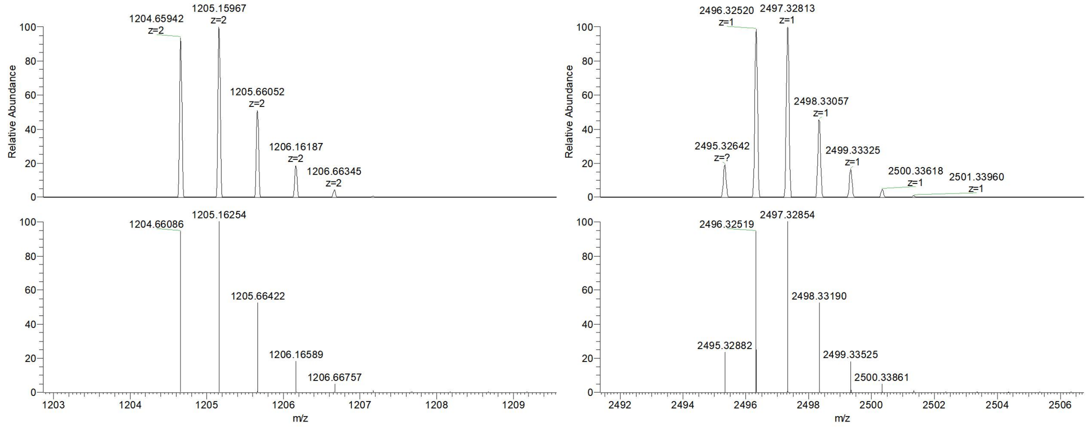

Supplementary Figure 29 ESI-MS spectrum of **1**.

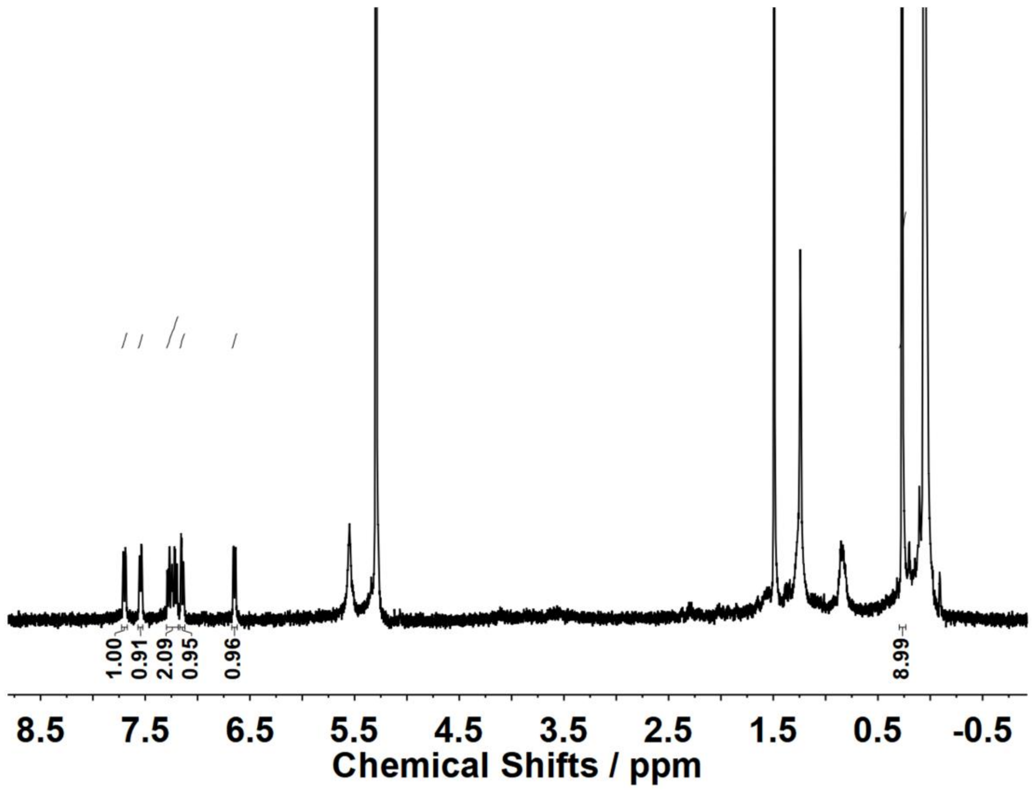

Supplementary Figure 30 ^{1}H NMR spectrum of **4** (400 MHz, CD_2Cl_2, 298K).

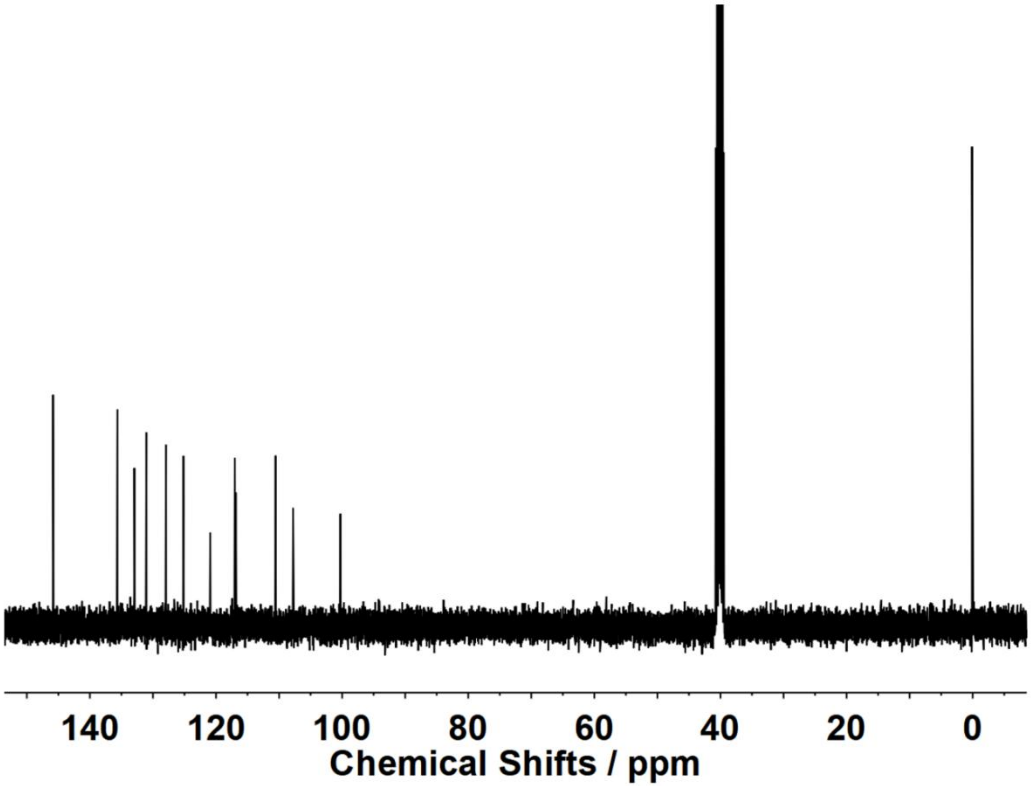

Supplementary Figure 31 ^{13}C NMR spectrum of **4** (100 MHz, d_6-DMSO, 298K).

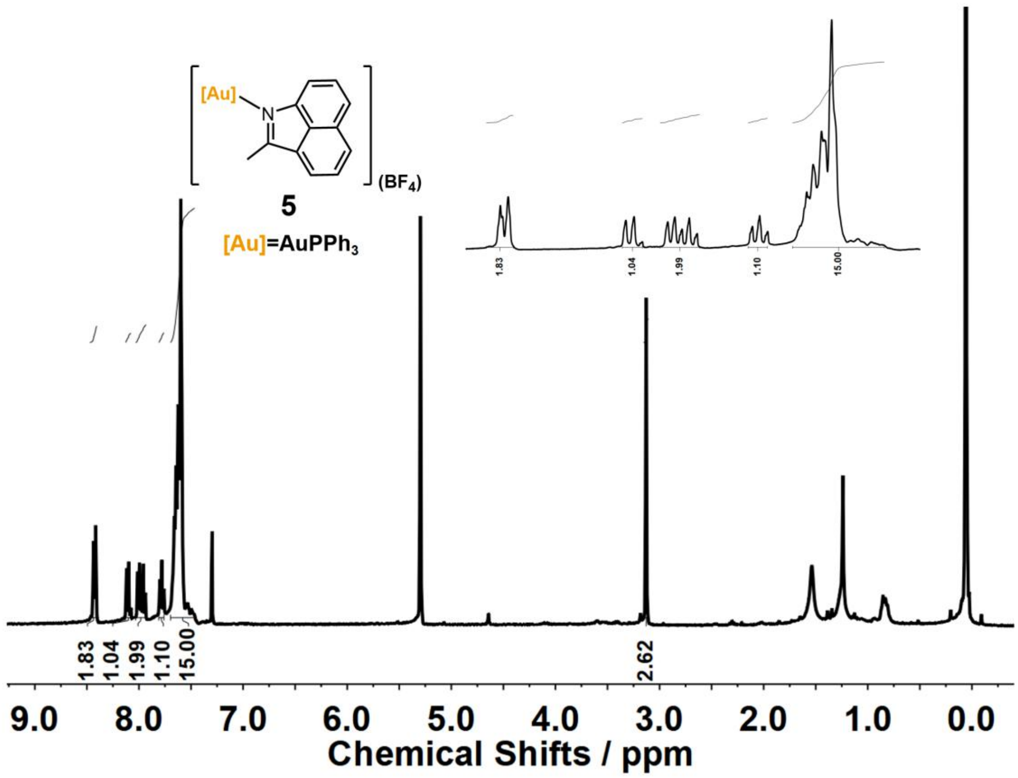

Supplementary Figure 32 ^{1}H NMR spectrum of **5** (400 MHz, CD_2Cl_2, 298K).

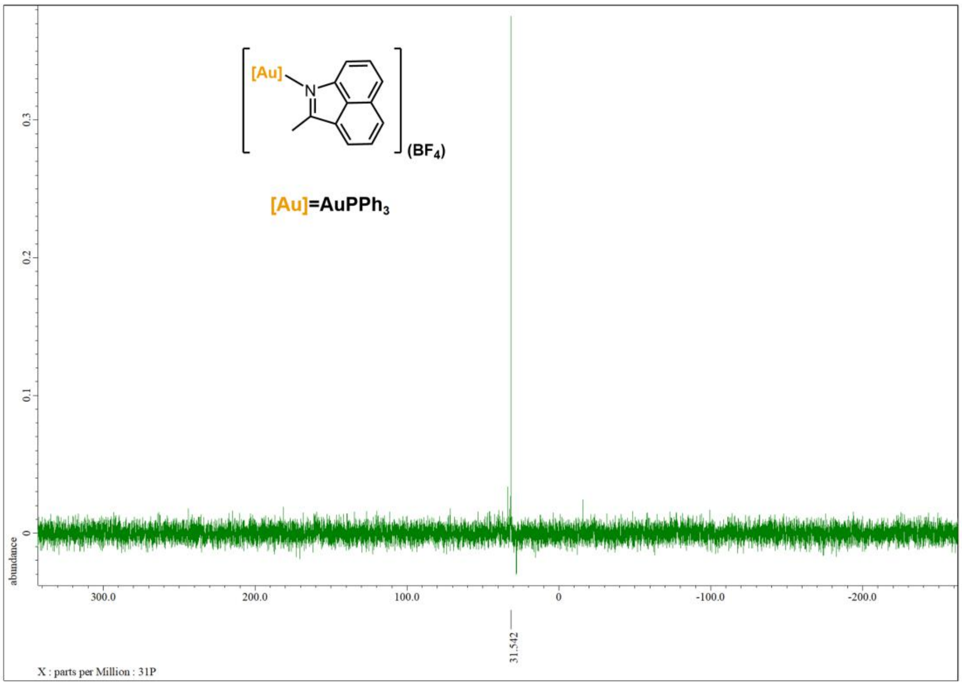

Supplementary Figure 33 ^{31}P NMR spectrum of **5** (162 MHz, CD_2Cl_2, 298K).

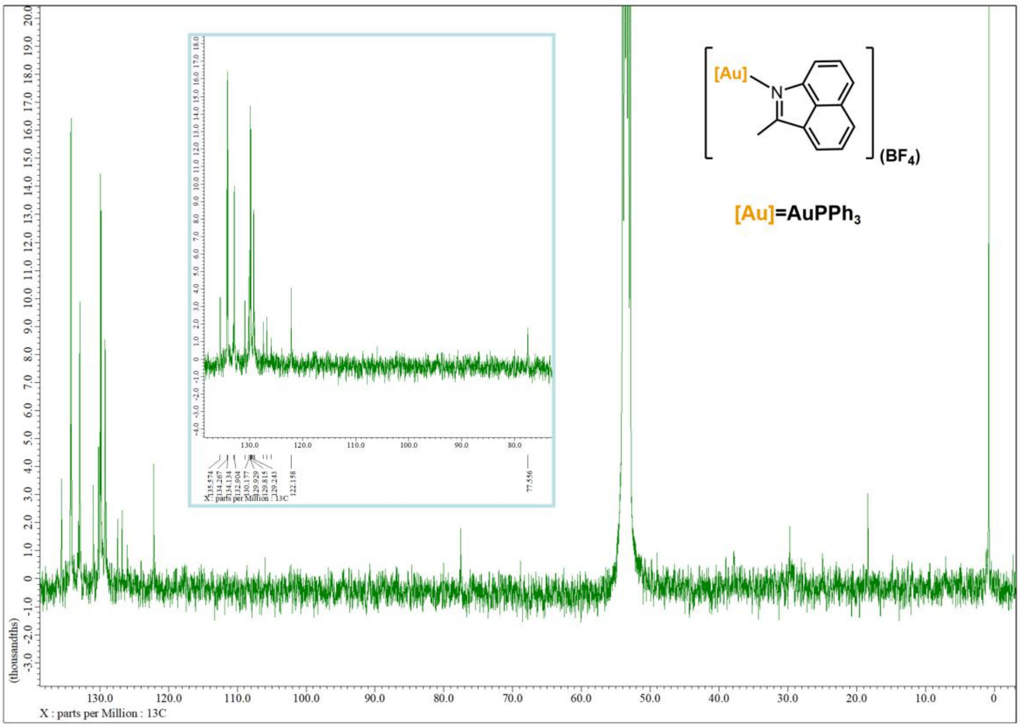

Supplementary Figure 34 ^{13}C NMR spectrum of **5** (100 MHz, CD_2Cl_2, 298K).

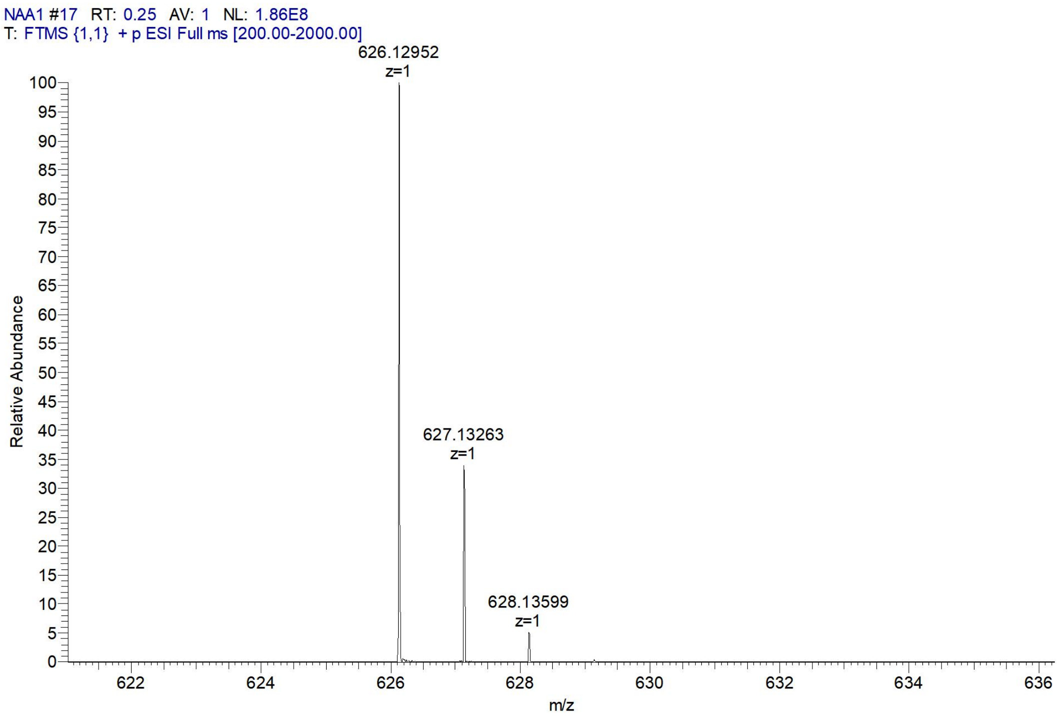

Supplementary Figure 35 ESI-MS spectrum of **5**.

Supplementary Tables

Supplementary Table 1. Calculated aromaticity data of **1-PMe$_3$** and **2-PMe$_3$**. Calculated NICS values (ppm), ΔBL (Å), EDDB_F(r) (e) and MCI of **1-PMe$_3$** and **2-PMe$_3$**.

	**1-PMe$_3$**	**2-PMe$_3$**
NICS(1)$_{zz}$ (ppm)	-24.5/-20.9	-26.4/-18.2
ΔBL (Å)	0.042/0.023	0.026/0.043
ΔBV(ELF$_\pi$)	0.283/0.222	0.177/0.544
EDDB_F(r) (e)	3.770/2.663	4.419/2.085

Supplementary Table 2. Calculated bonding analysis of **1-PMe3** and **2-PMe3**. Calculated multi-center bond order analysis (MCI), basin analysis of ELF(e) and Mayer bond order of **1-PMe3** and **2-PMe3**.

		**1-PMe3**	**2-PMe3**
MCI	C1-Au2	-	-
	Au2-C1-Au3	0.094	-
Basin analysis of ELF (e)	C1-Au2	-	2.28
	Au2-C1-Au3	2.61	-
Mayer bond order	N-Au1	0.77	0.82
	C1-Au2	0.70	1.02
	C1-Au3	0.63	-
	Au2-Au3	0.56	-

Supplementary Table 3. Fluorescence quantum yield of **4** (methods are according to the previous works[2]).

	Absorbance (A)	Emission Intensity[d] (E)	Refractive Index (n)	Quantum Yield (Q)
Quinine λ_{ex} = 340nm	0.04417[a]	70924	1	0.55
Quinine λ_{ex} = 350nm	0.04508[b]	71817	1	0.55
**4** λ_{ex} = 350nm	0.03705[c]	40378[e]	1.424[f]	0.76[g]<br>0.76[h]

[a] absorbance at 340nm. [b] absorbance at 350nm. [c] absorbance at 350nm. [d] integration of emission intensity. [e] the average of three times of measurements. [f] the refractive index of dichloromethane. [g] calculated by the data of Quinine at λ_{ex} = 340nm. [e] calculated by the data of Quinine at λ_{ex} = 350nm) [Quinine] = [**4**] = 5 μM.

Supplementary Methods

X-ray Crystallographic Analysis

Crystal data for **$[C_8H_4N(AuPPh_3)_5](BF_4)_2{\cdot}(CHCl_3)$** (**1**) (CCDC-1922291): $C_{99}H_{80}Au_5B_2Cl_3F_8NP_5$, M = 2703.29, monoclinic, P2(1)/c (No. 14), a = 16.313(1) Å, b = 16.664(1) Å, c = 34.377(1) Å, β = 99.32(1)°, V = 9221.8(2) Å^3, Z = 4, T = 100(1) K, D_c = 1.947 g/cm^{-3}, The structure, refined on F^2, converged for 14832 unique reflections (R_{int} = 0.0480) and 17421 observed reflections with $I > 2\sigma(I)$ to give R_1 = 0.0425 and wR_2 = 0.1120 and a goodness-of-fit = 1.038.

Crystal data for **$[C_8H_4N(AuPPh_3)_4](BF_4){\cdot}(C_2H_5OC_2H_5)$** (**2**) (CCDC-1922290): $C_{84}H_{74}Au_4BF_4NOP_4$, M = 2112.00, orthorhombic, Pbca (No. 61), a = 20.494(1) Å, b = 23.556(1) Å, c = 31.265(1) Å, V = 15093.5(3) Å^3, Z = 8, T = 173(1) K, D_c = 1.859 g/cm^{-3}, The structure, refined on F^2, converged for 11750 unique reflections (R_{int} = 0.0611) and 15131 observed reflections with $I > 2\sigma(I)$ to give R_1 = 0.0691 and wR_2 = 0.1938 and a goodness-of-fit = 1.064. Two fluorine atoms F3, F4 of the tetrafluoroborate anion are disordered at two positions with a defined occupancy ratio of 0.50:0.50.

Crystal data for **$[C_{12}H_9N(AuPPh_3)](BF_4){\cdot}(CHCl_3)$** (**5**) (CCDC-1936578): $C_{31}H_{25}AuBCl_3F_4NP$, M = 832.61, triclinic, P-1 (No. 2), a = 10.426(1) Å, b = 16.726(1) Å, c = 18.446(1) Å, α = 83.21(1)°, β = 75.26(1)°, γ = 82.20(1)°, V = 3070.2(1)Å^3, Z = 4, T = 107(1)K, D_c = 1.801 g/cm^{-3}, The structure, refined on F^2, converged for 10257 unique reflections (R_{int} = 0.0401) and 11602 observed reflections with $I > 2\sigma(I)$ to give R_1 = 0.0357 and wR_2 = 0.0977 and a goodness-of-fit = 1.026. Fluorine atoms in the tetrafluoroborate anion and chlorine atoms in the chloroform molecule are disordered at two positions with a defined occupancy ratio of 0.50:0.50.

Supplementary References

(1) Dempsey, C. E. Hydrogen Bond Stabilities in the Isolated Alamethicin Helix: pH–Dependent Amide Exchange Measurements in Methanol. *J. Am. Chem. Soc.* **117**, 7526–7534 (1995).

(2) Takaesu, N. A., Ohta, E., Zakharov, L. N., Johnson, D. W. & Haley, M. M. Synthesis and Properties of Naphtho[2,3–e]–1,2–azaphosphorine 2–Oxides: PN-Anthracene Analogues. *Organometallics* **36**, 2491–2493 (2017).
