# Supplementary material for: Hyperconjugative aromaticity and protodeauration reactivity of polyaurated indoliums
Source: Nat Commun. 2019 Dec 10;10:5639. doi: 10.1038/s41467-019-13663-8 (PMC6904676; doi:10.1038/s41467-019-13663-8)
Supplement: Supplementary file 2 — Description of Additional Supplementary Files [file 41467_2019_13663_MOESM2_ESM.pdf]

Description of Additional Supplementary Files

File Name: Supplementary Data 1

Description: Geometrical coordinates of the model complexes used for DFT calculation.
