# Supplementary material for: Hyperconjugative aromaticity and protodeauration reactivity of polyaurated indoliums
Source: Nat Commun. 2019 Dec 10;10:5639. doi: 10.1038/s41467-019-13663-8 (PMC6904676; doi:10.1038/s41467-019-13663-8)
Supplement: Supplementary file 3 — Supplementary Data 1 [file 41467_2019_13663_MOESM3_ESM.pdf]

Cartesian coordinates

1-PMe$_3$
SCF = -3346.34958743
2 1

Au	-3.29377000	0.17113600	1.10012200
Au	-0.51521900	1.30726500	-0.92572600
Au	2.24586600	1.46084600	0.51913600
Au	2.25356300	-1.41380400	0.53513400
Au	-0.60408900	-1.52290900	-0.66287300
C	-6.57733900	0.99808200	1.68691700
C	-5.73274000	1.23706400	-1.09564300
C	-6.20220900	-1.39128800	0.06144400
C	-1.19988200	4.54834800	-2.00397900
C	0.59191600	3.05622900	-3.75792800
C	-2.25121700	2.46217200	-3.72554600
C	4.97286500	3.52275300	1.05033200
C	2.83058200	4.78351800	-0.44654800
C	4.55965400	2.83679500	-1.74696700
C	5.47540100	-2.58953100	-0.07426500
C	3.51946100	-3.43170400	-2.04716000
C	3.55132700	-4.66124000	0.59536100
C	-2.92235900	-3.67156800	-2.04748000
C	-0.45086700	-4.96558800	-1.25118100
C	-0.53472400	-3.35225300	-3.67775000
C	-0.33023200	0.03770900	0.74343100
C	0.97151000	0.01600500	1.39519200
C	0.67830400	0.08528700	2.81534900
C	1.49010200	0.10180000	3.97114600
H	2.57453800	0.05380900	3.88032500
C	0.87386600	0.17955000	5.21574300
H	1.47917800	0.19246400	6.11980500
C	-0.54122500	0.24253000	5.33334300
H	-0.98584400	0.30312000	6.32468000
C	-1.36631600	0.22787300	4.21311300
H	-2.45054900	0.27592200	4.30159900
C	-0.74663600	0.14820900	2.95077600
N	-1.33851800	0.11749100	1.69859500
P	-5.45390800	0.24990300	0.43616000
P	-0.83446600	2.84601100	-2.60858000
P	3.64739700	3.15220100	-0.17495200
P	3.69489200	-3.03178300	-0.25461200
P	-1.10573900	-3.37940700	-1.92496900

H	-6.53988400	0.40846700	2.61022500
H	-6.24994900	2.01968600	1.91176500
H	-7.60842200	1.01933700	1.31127200
H	-5.40154900	2.26908500	-0.93020900
H	-5.15114100	0.80551800	-1.91901000
H	-6.79658700	1.23782100	-1.36569500
H	-6.15611800	-2.02672100	0.95341000
H	-7.24934700	-1.27292000	-0.24524800
H	-5.64060900	-1.87690900	-0.74528200
H	-0.37313000	4.90775300	-1.38091200
H	-1.34818300	5.23653900	-2.84622300
H	-2.10721500	4.52691000	-1.38883700
H	-3.17515500	2.41893700	-3.13696600
H	-2.35225500	3.23229400	-4.50129000
H	-2.09147100	1.48716100	-4.20066300
H	1.47537500	3.37114700	-3.19138400
H	0.81394500	2.09884400	-4.24371500
H	0.36549200	3.80909900	-4.52381300
H	-0.81590200	-5.10520400	-0.22693100
H	-0.77214500	-5.81258800	-1.87111900
H	0.64413100	-4.93168900	-1.22705600
H	-0.82972900	-4.27478100	-4.19439100
H	-0.97942500	-2.49174200	-4.19122000
H	0.55601200	-3.25003800	-3.71255800
H	-3.39457000	-2.81524900	-2.54335300
H	-3.13181300	-4.58397100	-2.62075500
H	-3.34167000	-3.77115800	-1.03936100
H	4.25304300	-4.18955500	-2.35091100
H	2.50971700	-3.81095800	-2.24081700
H	3.66991400	-2.52240700	-2.64102900
H	3.75083100	-4.53097100	1.66549000
H	2.53314600	-5.05095900	0.48130000
H	4.26719800	-5.38155300	0.17884600
H	5.69896500	-2.40134500	0.98231400
H	6.11622200	-3.40189700	-0.44101300
H	5.68483000	-1.67677100	-0.64433500
H	3.56796700	5.54513500	-0.73148300
H	2.08244200	4.69203100	-1.24197900
H	2.32409200	5.09571900	0.47423000
H	5.17785000	1.93861300	-1.63280200
H	3.84897100	2.66640700	-2.56425800
H	5.20463800	3.68872700	-1.99849200
H	5.61240100	4.33917100	0.69058700
H	4.51778800	3.81157600	2.00491700

H	5.58375200	2.62707100	1.21265200

2-PMe$_3$
SCF = -2749.64201793 a.u.
1 1

Au	-1.88702900	0.70123200	1.43684300
Au	-1.77806300	0.82551300	-1.41264800
Au	0.22937300	-1.42315300	-0.01118100
Au	3.68041800	0.37612200	0.05873300
C	0.67091000	0.55201800	0.07349000
C	-0.45590900	1.46532900	0.10051400
C	-0.39619700	4.10913300	0.20154800
H	-1.47525100	4.26304700	0.20047900
C	2.43130000	3.70878700	0.20350100
H	3.50971400	3.54917900	0.20428300
C	0.48458100	5.19330500	0.24703000
H	0.09099300	6.20801200	0.28259300
C	0.13309800	2.80767500	0.15592900
C	1.54491000	2.62398200	0.15776100
C	1.88400400	4.99440300	0.24790700
H	2.54616700	5.85809100	0.28400600
N	1.84172400	1.24838900	0.10714600
P	-0.57029200	-3.58098800	-0.09787700
P	-3.38080800	-0.13594600	2.95513700
P	-3.13501000	0.16299000	-3.12976900
P	5.71228500	-0.59866300	-0.01282900
C	-3.81456100	1.00754200	4.33622700
H	-4.53376700	0.54056800	5.02203600
H	-2.90365000	1.27256100	4.88561200
H	-4.24764100	1.92632600	3.92363300
C	-5.02638400	-0.62546400	2.26923300
H	-5.49993400	0.24926600	1.80777200
H	-4.88464000	-1.38855600	1.49417400
H	-5.68295600	-1.02274400	3.05490500
C	-2.81472300	-1.66661800	3.82132200
H	-2.63870200	-2.45520900	3.07979500
H	-1.86914200	-1.46139400	4.33658100
H	-3.56160600	-2.01209600	4.54836000
C	5.73137500	-2.24755700	-0.83956400
H	6.74543100	-2.66837100	-0.85095400
H	5.06048800	-2.92984800	-0.30411300
H	5.36981100	-2.14104400	-1.86897600
C	6.98425300	0.37590600	-0.92394500
H	7.10556500	1.35410600	-0.44419400

H	7.94812100	-0.14983400	-0.92749600
H	6.65112800	0.53537200	-1.95601200
C	6.48459400	-0.91317100	1.63168100
H	5.83230100	-1.56676100	2.22251600
H	7.46865200	-1.38592200	1.51571400
H	6.59782500	0.03692200	2.16666100
C	0.08598500	-4.67563500	-1.43265400
H	1.16650200	-4.80409000	-1.29700700
H	-0.40157800	-5.65941000	-1.41564100
H	-0.08262600	-4.20117100	-2.40677800
C	-2.39720500	-3.61503500	-0.38136200
H	-2.89058100	-3.01897100	0.39557800
H	-2.61461200	-3.15321600	-1.35167500
H	-2.79075000	-4.64001500	-0.36827500
C	-0.35792900	-4.59846300	1.42927700
H	-0.82152300	-5.58753700	1.31609300
H	0.71168800	-4.72038700	1.63737100
H	-0.81262700	-4.07688600	2.27978000
C	-2.39089200	-1.10049200	-4.25379200
H	-1.47475400	-0.69217900	-4.69631400
H	-2.12201200	-1.99135700	-3.67315600
H	-3.08925500	-1.38271200	-5.05286600
C	-4.73973400	-0.60464600	-2.62713100
H	-5.31353300	0.11773600	-2.03441400
H	-5.32960100	-0.90725500	-3.50254500
H	-4.54257000	-1.48319400	-2.00100000
C	-3.64406500	1.51668100	-4.27471400
H	-4.28126500	1.13355500	-5.08285600
H	-4.19122000	2.28106000	-3.71037100
H	-2.74898000	1.98122100	-4.70471400

Furan
SCF = -230.067321977 a.u.
0 1

C	-0.00000000	-1.15433719	0.00000000
C	1.13714965	-0.39491029	-0.00000000
C	0.70378335	0.97644465	-0.00000000
C	-0.66342173	0.94483698	0.00000000
O	-1.11453085	-0.35213047	0.00000000
H	-0.19878381	-2.21700437	0.00000000
H	2.15740229	-0.75959871	-0.00000000
H	1.32927851	1.86108573	-0.00000000
H	-1.43671779	1.70035614	0.00000000

Indol

SCF = -363.902789539 a.u.

0 1

N	1.10224260	1.55800870	0.00000000
C	2.26379661	0.79738955	0.00000000
C	1.93331231	-0.53970608	0.00000000
C	0.49682569	-0.62371907	0.00000000
C	-0.43119840	-1.68667932	0.00000000
H	-0.08462511	-2.71950946	0.00000000
C	-1.79519253	-1.39598128	0.00000000
H	-2.51888241	-2.20990164	0.00000000
C	-2.26047731	-0.05965337	0.00000000
H	-3.33208411	0.13402556	0.00000000
C	-1.37091135	1.01624492	0.00000000
H	-1.72932934	2.04510491	0.00000000
C	0.00000000	0.71856434	0.00000000
H	3.23209515	1.28291182	0.00000000
H	2.63289258	-1.36712756	0.00000000
H	1.06730503	2.56967728	0.00000000

2H-indolium

SCF = -364.240000122 a.u.

1 1

C	-0.18376172	-0.64038799	0.00003421
C	-0.18917045	0.76322776	0.00005138
C	1.03733535	1.43981370	0.00002928
C	2.21298027	0.67315757	0.00001928
C	2.17583546	-0.73145890	-0.00000543
C	0.95267918	-1.43032384	-0.00002823
C	-2.41953608	0.17191939	0.00014288
C	-1.57919731	1.22160087	-0.00011630
H	1.07771210	2.52614371	-0.00000785
H	3.17635490	1.17727935	-0.00000968
H	3.10488062	-1.29589686	-0.00003625
H	0.92275833	-2.51757873	-0.00007092
H	-1.89726440	2.25888229	-0.00027202
H	-3.49721111	0.07526721	0.00002317
H	-1.82627363	-1.67467846	-0.83068940
H	-1.82629061	-1.67478732	0.83053956
N	-1.61109348	-1.09713180	-0.00003414

2Au-indolium

SCF = -1556.97527436 a.u.

1 1

Au	1.49549226	-0.17135365	-0.20879517

Au	-1.49868855	-0.16431486	-0.20857832
N	0.00140936	1.25051819	-0.64843599
C	0.00286365	1.75449017	-1.99357339
C	0.00723397	3.11963428	-2.00102526
C	0.00878601	3.57111952	-0.62598371
C	0.01281986	4.83997023	-0.01291044
H	0.01569493	5.74610042	-0.61578958
C	0.01322753	4.91055256	1.38157388
H	0.01643349	5.88339244	1.86920257
C	0.00960224	3.74242195	2.17683844
H	0.00999430	3.83193365	3.26130574
C	0.00545344	2.47269110	1.59043120
H	0.00261955	1.56438279	2.19355194
C	0.00517707	2.41246510	0.19223803
H	0.00044083	1.04600596	-2.81339283
H	0.00915133	3.74812733	-2.88471005
P	3.14152634	-1.64329452	0.27342678
P	-3.14655732	-1.63388266	0.27400639
C	-4.22205024	-1.08528266	1.66295967
H	-4.68451352	-0.12462223	1.40880899
H	-5.00661669	-1.82825403	1.85654770
H	-3.61602511	-0.95207321	2.56660427
C	-2.54988695	-3.30412932	0.77110671
H	-1.92043105	-3.21520196	1.66421373
H	-3.39786828	-3.96591847	0.98982416
H	-1.95212244	-3.73615128	-0.03997040
C	-4.29809558	-1.94045124	-1.12801181
H	-5.08208235	-2.64876142	-0.83061120
H	-4.75881018	-0.99441123	-1.43464615
H	-3.74160252	-2.34951762	-1.97913555
C	2.54870111	-3.24914365	0.95401879
H	3.39719414	-3.91003186	1.17335550
H	1.98374122	-3.06763727	1.87574511
H	1.88871146	-3.73509934	0.22598740
C	4.33078819	-1.01140527	1.52771238
H	5.11525872	-1.75460205	1.72080750
H	4.78829319	-0.08483697	1.16235787
H	3.79866425	-0.79297401	2.46081597
C	4.17634378	-2.09716856	-1.17915654
H	4.63164513	-1.19264546	-1.59850762
H	4.96640886	-2.79863132	-0.88143370
H	3.54814228	-2.56131560	-1.94817950
